# Extensive remodeling of sugar metabolism through gene loss and horizontal gene transfer in a eukaryotic lineage

**DOI:** 10.1186/s12915-024-01929-7

**Published:** 2024-05-30

**Authors:** Ana Pontes, Francisca Paraíso, Margarida Silva, Catarina Lagoas, Andreia Aires, Patrícia H. Brito, Carlos A. Rosa, Marc-André Lachance, José Paulo Sampaio, Carla Gonçalves, Paula Gonçalves

**Affiliations:** 1 UCIBIO, Department of Life Sciences, NOVA School of Science and Technology, Universidade NOVA de Lisboa, Caparica, Portugal; 2 Associate Laboratory i4HB, NOVA School of Science and Technology, Universidade NOVA de Lisboa, Caparica, Portugal; 3 PYCC – Portuguese Yeast Culture Collection, Department of Life Sciences, NOVA School of Science and Technology, Universidade NOVA de Lisboa, Caparica, Portugal; 4 Departamento de Microbiologia, ICB, C.P. 486, Universidade Federal de Minas Gerais, Belo Horizonte, MG 31270-901 Brazil; 5 Department of Biology, University of Western Ontario, London, ON N6A 5B7 Canada

**Keywords:** Gene loss, Horizontal gene transfer, Yeast metabolism, Yeast genome evolution, W/S clade

## Abstract

**Background:**

In yeasts belonging to the subphylum Saccharomycotina, genes encoding components of the main metabolic pathways, like alcoholic fermentation, are usually conserved. However, in fructophilic species belonging to the floral *Wickerhamiella* and *Starmerella* genera (W/S clade), alcoholic fermentation was uniquely shaped by events of gene loss and horizontal gene transfer (HGT).

**Results:**

Because HGT and gene losses were first identified when only eight W/S-clade genomes were available, we collected publicly available genome data and sequenced the genomes of 36 additional species. A total of 63 genomes, representing most of the species described in the clade, were included in the analyses. Firstly, we inferred the phylogenomic tree of the clade and inspected the genomes for the presence of HGT-derived genes involved in fructophily and alcoholic fermentation. We predicted nine independent HGT events and several instances of secondary loss pertaining to both pathways. To investigate the possible links between gene loss and acquisition events and evolution of sugar metabolism, we conducted phenotypic characterization of 42 W/S-clade species including estimates of sugar consumption rates and fermentation byproduct formation. In some instances, the reconciliation of genotypes and phenotypes yielded unexpected results, such as the discovery of fructophily in the absence of the cornerstone gene (*FFZ1*) and robust alcoholic fermentation in the absence of the respective canonical pathway.

**Conclusions:**

These observations suggest that reinstatement of alcoholic fermentation in the W/S clade triggered a surge of innovation that goes beyond the utilization of xenologous enzymes, with fructose metabolism playing a key role.

**Supplementary Information:**

The online version contains supplementary material available at 10.1186/s12915-024-01929-7.

## Background

Comparative genomics combined with increasingly sophisticated molecular genetics tools and high-throughput microbial physiology studies set the stage for the present era of exciting discoveries concerning the evolution of microbial metabolism. For yeasts belonging to the subphylum Saccharomycotina, this was potentiated by the early availability of an appreciable number of genome sequences and more recently by the publication of broad range studies based on high-throughput data and analyses made available to the scientific community [1–4]. Amidst the achievements made possible by the newly available resources, we may count the discovery of new genetic code alterations in two yeast lineages [5], the impact of partial loss of DNA repair genes in the evolution of *Hanseniaspora* [6] or the finding of an unusually high number of genes acquired through horizontal gene transfer (HGT) in the *Wickerhamiella/Starmerella* clade (henceforward named the W/S clade) [7, 8]. The latter clade turned out to be an outstanding model for the study of the evolution of metabolism, because a significant portion of the genes already identified as having been acquired via HGT encode metabolic enzymes, some of which are functional and contribute to the metabolic toolkit of the recipient yeast species [8–11]. The acquired genes originated in both the Pezizomycotina (ascomycetous filamentous fungi) [12] and various bacterial lineages [2, 8–10]. As more of these instances are characterized in detail, a remarkable picture develops, showing where and how incoming xenologs interacted with native gene networks.

The W/S-clade ancestor seems to have undergone a pronounced loss of metabolic genes and capabilities [2], among which one of the most emblematic yeast traits—alcoholic fermentation [8]. We previously showed for *Starmerella bombicola* and a handful of other W/S-clade species that alcoholic fermentation was reacquired making use of *ADH1* (alcohol dehydrogenase) genes of bacterial origin, together with the recruitment of an alternative enzyme to replace pyruvate decarboxylase (Pdc) [8]. The latter enzyme catalyzes the first step of alcoholic fermentation, converting pyruvate into acetaldehyde while the former catalyzes the second and final step consisting in the reduction of acetaldehyde to yield ethanol. As far as it was possible to ascertain, all Saccharomycotina yeasts possess native *PDC* and *ADH1* vertically inherited genes, except those included in the W/S clade [8].

Another metabolic hallmark of the W/S clade is the fact that most species are fructophilic, i.e., they prefer fructose to glucose as a carbon source when both sugars are present in the growth medium at high concentrations. A highly unusual fructose transporter (*FFZ1*) [12] was shown to be the cornerstone of fructophily in yeasts, not only in the W/S clade but also in the genus *Zygosaccharomyces*, which apparently received *FFZ1* from a species in the W/S clade, also through HGT [12]. Concerning the possible advantage conferred by the assimilation of fructose *vs* glucose, it was shown for *St. bombicola* that a considerable fraction of the fructose consumed was converted directly to mannitol [13]. Together with the observation that mannitol production is unusually frequent among W/S-clade species, we hypothesized that in the W/S-clade ancestor mannitol formation may have served as an alternative to restore redox balance in the absence of alcoholic fermentation [13]. This constitutes a plausible link between acquisition of fructophily and loss of alcoholic fermentation and resembles the proposed hypothesis for the evolution of fructophily in bacteria [14–18]. Fructophilic bacteria are a group of lactic acid bacteria that thrive in the floral environment and independently evolved fructophily, possibly triggered by the loss of alcoholic fermentation [15, 19, 20]. Absence of alcoholic fermentation is thought to negatively impact redox balance when glucose is used as the sole energy source, due to an impairment of NAD(P)^+^ regeneration [16]. Contrary to glucose, fructose can be used as an electron acceptor with mannitol as the final product, regenerating the needed oxidized cofactors [15, 17]. In the context of these observations and hypothesis, we note that mannitol and glycerol production may be considered alternative fermentations in the sense that their formation recycles oxidized co-factors (NAD^+^ or NADP^+^) and that the byproducts extruded are likely to have an ecological impact on the microbial community, which are two important features in common with alcoholic fermentation.

The large number of remarkable findings already reported for the evolution of sugar metabolism in the W/S clade included the loss of alcoholic fermentation in the MRCA of the clade and subsequent reinstatement through four independent events of acquisition of bacterial *ADH1*, *ADH6*, and *PDC1* genes [8] and also a single acquisition of fructophily-associated transporter gene *FFZ1* from filamentous fungi by the W/S-clade MRCA [12]. However, previous studies were based on the analyses of very few (< 10) genomes, which can impact the resolution of evolutionary inferences due to incomplete taxon sampling [21]. Here, we revisit the evolutionary events associated with fructophily and fermentation based on the analysis of an expanded dataset of 63 genomes representing the majority of the species currently recognized in the clade and on the phenotypic characterization of 42 strains across the clade. This characterization consisted mainly in measuring the amount and rate of ethanol production by the various species, but we also included the quantification of mannitol and glycerol production, when present. Concerning the evolutionary occurrences, we inferred a total of eight putative HGT events involved in the acquisition of alcoholic fermentation-related genes, including three different types of bacterial *ADH1* genes. However, we found that although most *Starmerella* and *Wickerhamiella* species harbor a bacterial *ADH1* gene, alcoholic fermentation is only pervasive in a monophyletic lineage that we refer to as the *Starmerella* subclade. Our results highlighted a phenotypic landscape in which different W/S-clade species use a broad range of fermentation *vs* respiration ratios, rather than falling into discrete respiratory or fermentative categories. Moreover, the reconciliation of phenotypes and genotypes in a subset of strains selected across the entire clade led to the discovery of cases of fructophily in the absence of the cornerstone gene *FFZ1* and alcoholic fermentation taking place in the absence of the genes normally composing the respective canonic pathway. Finally, we put forward a refined hypothesis concerning the evolutionary links between fructophily and alcoholic fermentation. Our results underscore the relevance of contrasting evolutionary mechanisms (gene loss and HGT) in the evolution of adaptive traits in yeasts and illustrate how trait loss may be a driver of metabolic innovation.

## Results and discussion

### An expanded phylogenomic tree of the W/S clade

**Fig. 1:**
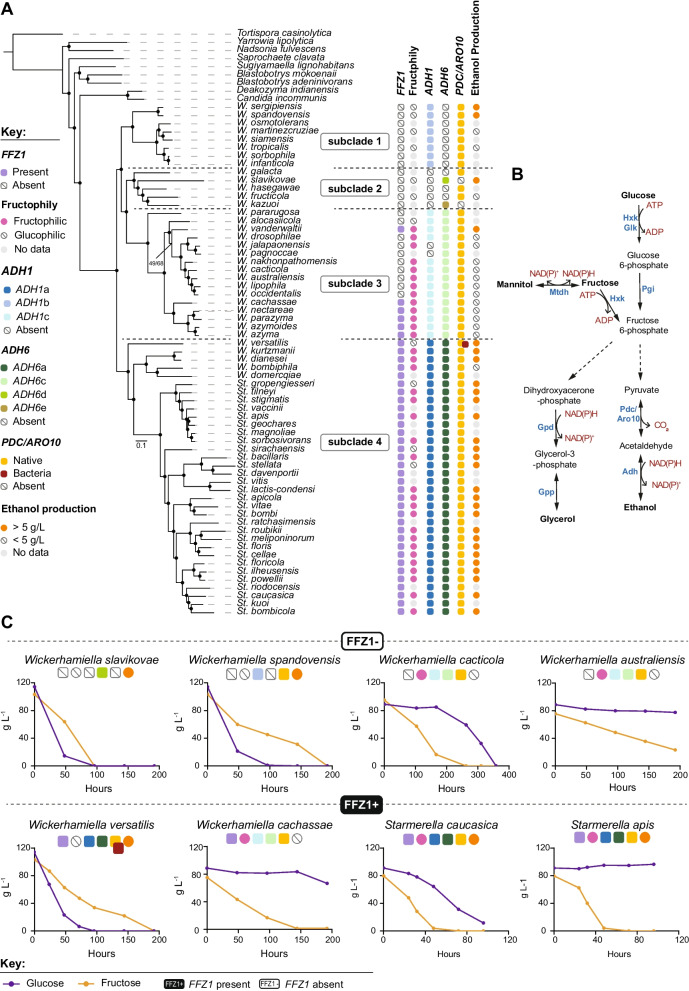
Phylogenomic tree of the W/S clade and distribution of fructophily and fermentation-related genes and phenotypes. **A** Maximum likelihood phylogenomic tree comprising 63 W/S-clade species and nine outgroups, inferred from the concatenated alignment of 264 SCO and rooted with *Tortispora caseinolytica* based on the phylogenetic analysis of Shen et al. 2018 [2]. The presence/absence of genes related to fructophily and alcoholic fermentation, as well as phenotypes determined for a subset of species, are shown to the right of the tree. Subclades (labeled 1–4) were defined according to the *ADH1* genotype. **B** Relevant metabolic pathways for production of fermentation byproducts in the W/S clade (mannitol, glycerol, and ethanol). Names of the enzymes carrying out the represented reactions are shown next to the respective arrow: Hxk—hexokinase, Glk—glucokinase, Pgi—phosphoglucoisomerase, Mtdh—mannitol dehydrogenase, Pdc—pyruvate decarboxylase, Aro10—phenylpyruvate decarboxylase, Adh—alcohol dehydrogenase, Gpd—glycerol 3-phosphate dehydrogenase, Gpp—glycerol 3-phosphate phosphatase. **C** Sugar consumption profiles for eight W/S-clade species cultivated in medium containing glucose and fructose (20FG) are shown, highlighting the diversity in sugar consumption rates and preferences across the W/S clade. For each species, the presence/absence profile of genes and traits depicted in **A** is shown above the plots. Sugar consumption profiles and fermentation byproduct formation profiles all species as well as respective replicates are shown in Additional file 5: Fig. S4

To shed light onto the evolution of fructophily and alcoholic fermentation, we expanded the number of species in the W/S clade for which whole-genome data is available (Additional file 1: Table S1). Raw data were assembled and annotated using the same pipelines for all species, irrespective of the origin of the data. The resulting phylogenomic tree encompasses 72 species (63 in the W/S clade and nine outgroups) and is based on 273 single-copy orthogroups (SCO) present in all species (Fig. 1). Its topology confirmed previous observations indicating that several *Wickerhamiella* species cluster robustly with species currently assigned to the genus *Starmerella* (subclade 4) [7]. These include the type species of the genus, *Wickerhamiella domercqiae*. For this reason and others that will become apparent, we shall refer to these (five) *Wickerhamiella* species together with all *Starmerella* species as the *Starmerella* subclade (subclade 4) and to the remaining species as the *Wickerhamiella* subclade (comprising subclades 1, 2, and 3). This topology is not a consequence of the fact that many, possibly all, W/S-clade species have numerous genes acquired horizontally from bacteria and from filamentous fungi (Pezizomycotina), since it is very unlikely that any such genes were represented in the set of SCO used to infer the phylogeny; only SCO present in all species were considered, and HGT-derived genes tend to be present only in a fraction of the taxa [8–11]. The expanded number of species included in this tree produced a more complex topology within both the *Wickerhamiella* and *Starmerella* subclades, thereby improving the resolution of the framework onto which previously identified evolutionary events can be mapped.

### Multiple independent HGT events involving alcoholic fermentation-related genes

The distribution and phylogenetic relationships of alcoholic fermentation-related genes across the W/S clade show three clearly separated groups in the *ADH1* phylogeny (Additional file 2: Fig. S1) and four in the *ADH6* phylogeny (Additional file 3: Fig. S2), suggesting that seven independent HGT events were involved in the acquisition of *ADH1*-like (total of three events) and *ADH6*-like genes (total of four events).

*Starmerella bombicola* was previously shown to harbor one *ADH1*-like and two *ADH6*-like genes [8]. Considering the entire W/S clade, many species have similarly one *ADH1* gene, with some harboring two and *W. versatilis* containing three *ADH1* genes (Fig. 1). *ADH6* genes are almost always present in multiple copies ranging in number from two to ten. Phylogenetic analyses of the *ADH1* and *ADH6* genes found in W/S-clade species show that whenever more than one gene is present, duplications occurred after speciation, as multiple *ADH1* or *ADH6* genes present in any given species always clustered together (Additional file 2: Fig. S1, Additional file 3: Fig. S2). The distinct bacterial origin of the *ADH* genes, suggesting multiple independent acquisitions, is remarkably consistent with the topology of the phylogenomic tree (Fig. 1). For *ADH1*, three different bacterial origins were identified in three different subclades. All species in the *Starmerella* subclade (subclade 4) possess *ADH1* genes from Acetobacteraceae (named Adh1a). In subclade 1, all species possess a *ADH1* gene (named Adh1b) possibly derived from the Enterobacterales, while in subclade 3, all but two species have an *ADH1* gene possibly originating in *Acinetobacter* (named Adh1c). The two exceptions are *W. pagnoccae* and *W. jalapaonensis*, both lacking *ADH1* genes, probably as consequence of a secondary loss in their common ancestor. Finally, subclade 2 contains five species all seemingly lacking *ADH1* genes. Plotting the distribution of the genes onto the phylogenomic tree, we postulate that the three types of *ADH1* genes found in *Wickerhamiella* and *Starmerella* species were each independently acquired by the ancestor of the subclade in which they are presently found while subclade 2 seems never to have acquired a bacterial *ADH1* gene.

A more complicated pattern emerges for *ADH6* genes, where four different bacterial genes seem to have been acquired by three different subclades, as inferred from the distribution of *ADH1.* Specifically, subclade 4 harbors Adh6a enzymes possibly originating in the *Sphingomonadales* whereas most species of subclade 3 contain multiple *ADH6*c genes possibly originating in the *Alteromonadales*. However, subclade 1 seems to lack *ADH6* genes, while two species in the Adh1null subclade 2 contain *ADH6* genes that seem to be of different bacterial origins (Adh6d and Adh6e).

### W/S-clade species harboring different bacterial alcohol dehydrogenases exhibit distinct fermentative phenotypes

We showed previously that in *St. bombicola* Adh1a was mainly responsible for alcoholic fermentation but also ensured ethanol assimilation [8]. By contrast, Adh6 enzymes in *St. bombicola* are unconnected with alcoholic fermentation, as in *Saccharomyces cerevisiae*, although they might make a minor contribution to this process in the absence of Adh1 [8]. This agrees with the fact that in most if not all species studied so far, the interconversion of acetaldehyde and ethanol is ensured by a NADH-dependent Adh1 type of enzyme [22–24], while Adh6-type enzymes are deemed to be broad range, NADPH-dependent enzymes that accept a variety of aldehydes as substrates (Fig. 1B) [25, 26]. In conformity with this, all species tested in the *Starmerella* subclade (subclade 4) conducted alcoholic fermentation except for *W. bombiphila* (Fig. 1). However, almost without exception, species encoding Adh1b or Adh1c types of enzymes seemed to be poor ethanol producers (< 5 g/L) under the conditions tested. Unlike Adh1a, these enzymes may be involved mainly in ethanol assimilation (Additional file 1: Table S1). If that is the case, Adh1b and Adh1c would functionally resemble *S. cerevisiae* Adh2, an enzyme specialized in ethanol assimilation and that does not participate in the inverse reaction [27]. Nevertheless, we found two exceptions in subclade 1, the closely related species *W. spandovensis* and *W. sergipiensis*, which produce ethanol in substantial amounts, presumably using Adh1b. The most intriguing case is, however, *W. slavikovae* which is capable of robust fermentation while presumably lacking both Adh1 and Aro10, the enzyme deemed to have replaced Pdc in alcoholic fermentation in the W/S clade [8]. Absence of fermentation genes in *W. slavikovae* was confirmed by inspection of an additional high-quality assembly produced using long reads (Nanopore technology) (GCA_954870865). Even if we assume that one or more of the products of the five *ADH6* genes found in this species may be responsible for ethanol formation, the enzyme responsible for the conversion of pyruvate to acetaldehyde remains elusive. An alternative pathway for ethanol production might involve an AdhE type of multifunctional enzyme, commonly found in bacteria [28]. However, a gene encoding this kind of enzyme, which might fulfill the roles of both Adh1 and Pdc, was also absent from the *W. slavikovae* genome. One additional species, *W. kazuoi*, also lacks Pdc, Aro10, and Adh1. These observations indicate that the loss of Adh1 and Aro10 might be connected, suggesting that the two enzymes are functionally linked.

In summary, we identified at least seven independent events of acquisition of *ADH* genes, involving four W/S subclades. *ADH1* or *ADH6* genes were each acquired by the ancestor of a subclade, except for Adh6d and Adh6e, which are found each in a single species. The ancestors of subclades 1 and 4 acquired Adh1 and Adh6 at the same point in evolution but from different bacterial donors (Additional file 2: Fig. S1, Additional file 3: Fig. S2). In the *Starmerella* subclade (subclade 4), this formed the basis for a fermentative capacity that persisted in almost all species, while in the two *Wickerhamiella* subclades possessing Adh1 (subclades 1 and 3), ethanol assimilation might be the most likely role of the enzyme. As for Adh6, an involvement in alcoholic fermentation seems so far negligible, with the possible exception of Adh6d in *W. slavikovae*; a likely hypothesis may be that Adh6 play a role in detoxification, carrying out the conversion of aldehydes of environmental origin to the less toxic corresponding alcohols [29–31]. Whatever the role, multiple copies of bacterial *ADH6* genes are widespread across the entire W/S clade, while most species carry only one *ADH1* gene. As to the evolution of Pdc/Aro10, it is notable that only *W. versatilis* acquired bacterial Pdc genes (Additional file 4: Fig. S3), as previously reported [8]; no other bacterial Pdc genes were found in other W/S-clade species (Additional file 4: Fig. S3). It cannot be excluded that the inability of species in the *Wickerhamiella* subclades to conduct alcoholic fermentation is due to the absence of Pdc activity, which is not required for ethanol assimilation. In other words, it cannot be excluded that co-optation of Aro10 for alcoholic fermentation occurred only in a subset of W/S-clade species. At variance with this possibility, the very few *Wickerhamiella* species lacking Aro10 are among the few that also lack Adh1, including one likely to have experienced Adh1c secondary loss, suggesting a functional link between the two enzymes. Finally, we found a strong ethanol producer, *W. slavikovae* in the Adh1-null subclade (subclade 2), that also lacks Pdc/Aro10 (Fig. 1). Together, these observations suggest a complex evolutionary pattern involving Aro10 and its role in fermentation in the W/S clade. These observations also imply the existence of yet unidentified enzymes promoting ethanol production in substantial amounts in species lacking Adh1 and Pdc/Aro10, like *W. slavikovae.*

### The phenotypic landscape across the W/S clade reveals a wide range of fermentation rates

**Fig. 2:**
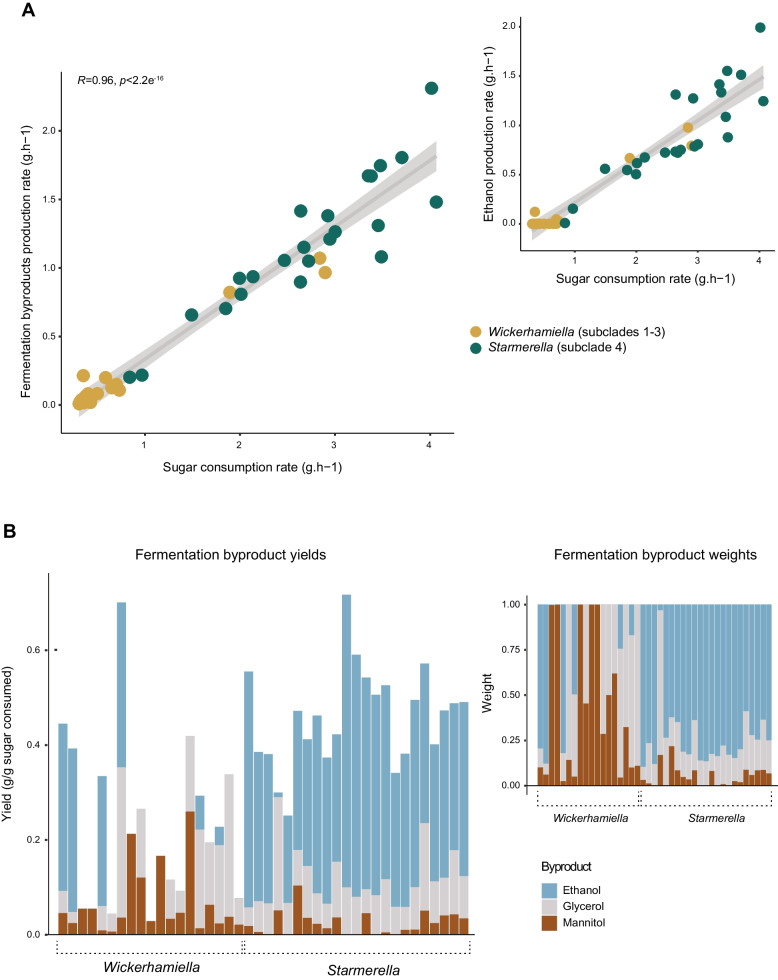
Comparison of respiration vs fermentation preferences across the W/S clade. **A** Correlation between maximum global fermentation byproduct production rates (ethanol, glycerol, and mannitol) and sugar consumption rates across the W/S clade, highlighting *Wickerhamiella* (subclades 1–3) and *Starmerella* (subclade 4) species in different colors. The inset (top, right) shows the correlation between sugar consumption rate and ethanol production rate determined at the same time points for the same species, denoting that the correlation is mainly driven by ethanol production. Each datapoint represents a single measurement for each species, but results for a replicate assay can be assessed in Additional file 6: Table S2 and Additional file 8: Fig S6A. **B** Fermentation byproduct (ethanol, glycerol, and mannitol) individual yields determined for each byproduct at the time point at which the maximum global yield was measured for each species (replicate assay can be assessed in Additional file 6: Table S2). The more pronounced bias towards fermentative metabolism in the *Starmerella* clade (subclade 4) when compared to *Wickerhamiella* (subclades 1–3) is noticeable. The manifest difference between *Starmerella* (subclade 4) and *Wickerhamiella* (subclades 1–3) concerning the main fermentation byproduct formed is highlighted in the inset (top, right) where the proportions of the three byproducts formed by each species are shown

The general trend observed in Fig. 1 is that *Starmerella* species conduct alcoholic fermentation while species in the *Wickerhamiella* subclades generally do not. However, the amount of ethanol (and other fermentation byproducts—mannitol and glycerol) (Fig. 1B) produced by fermentative species and their production rates vary considerably (Additional file 5: Fig. S4, Additional file 6: Table S2). The correlation between global (considering all products together) maximum fermentation byproduct production rates determined for 42 species studied and the extremely variable sugar consumption rates observed is shown in Fig. 2A. Not surprisingly, the rate of global fermentation byproduct formation robustly correlates with the sugar consumption rate measured using the same time point (*r* = 0.96, *p*-value < 2.2e^−16^, Pearson correlation test) (Fig. 2A), showing no major byproduct of fermentation was overlooked. Hence, as usually observed [32], sugars are consumed more rapidly during fermentative metabolism to compensate for the lower energetic yield of fermentation. Interestingly, we found that the phenotypic landscape of fermentation and sugar consumption rates covers a wide spectrum of values (Fig. 2A), indicating that the proportion of fermentative metabolism *vs* respiratory metabolism is extremely diverse across the clade under the conditions tested. Globally, the correlation between fermentation rates and sugar consumption is mainly driven by ethanol production (*r* = 0.95, *p*-value < 2.2e^−16^, Pearson correlation test), since, as shown on the inset of Fig. 2A, the correlation between sugar consumption rates and fermentation rates is barely affected when glycerol and mannitol are excluded (Fig. 2A, inset). We corrected these correlations for the effect of phylogenetic relatedness using PIC (phylogenetically independent contrasts) (Additional file 7: Fig. S5) and showed that the robust correlation is maintained in both cases (all fermentation byproducts: adjusted *R*-squared = 0.837, *p*-value < 2.2e^−16^; only ethanol: adjusted *R*-squared = 0.779, *p*-value < 6.62e^−15^). In line with our previous observations, the highest sugar consumption and fermentation rates are found in species belonging to the *Starmerella* subclade (subclade 4) (Fig. 2A, Additional file 8: Fig. S6). This is also noticeable when the absolute amounts of fermentation byproducts (in the form of individual byproduct yields) are compared between the two groups (subclade 4 and the remaining three *Wickerhamiella* subclades) (Fig. 2B). In *Wickerhamiella*, mannitol and glycerol account for a much larger fraction of the fermentation byproducts than in *Starmerella* (Fig. 2B), which is consistent with the observation that the global fermentation byproduct formation rates drop to zero in most of these species when only ethanol is considered (Fig. 2A, inset). Hence, *Starmerella* species resort almost without exception to alcoholic fermentation for energy conservation often with concomitant formation of mannitol and/or glycerol, while the majority of *Wickerhamiella* species use respiration and exhibit a much wider diversity concerning byproduct formation, ranging from considerable amounts of mannitol and/or glycerol to nearly none (Fig. 2B).

### Presence of HGT-derived FFZ1 is pervasive across fructophilic species

Possibly the most unusual trait that is widely spread in the W/S clade is the preference for fructose over glucose when both sugars are present in large amounts. The preference is often far from subtle as in some of the species studied so far, glucose consumption only picked up after a considerable drop in fructose concentration (Fig. 1C). The cornerstone of this marked fructophilic behavior is a remarkable high-capacity fructose transporter (Ffz1) that evolved from a Drug/H^+^ antiporter relatively recently, therefore lacking the usual signatures of sugar porters [12]. All the evidence indicates that *FFZ1* has been horizontally acquired from the Pezizomycotina early in the evolution of the W/S clade [12]. Initial research found an almost perfect correlation between the presence of *FFZ1* in the genome and fructophily [12, 33]. To find out whether this strong correlation persisted after the substantial increase in the size of the genome sequence dataset here reported, we scored the presence of the *FFZ1* gene in all species represented in the phylogenomic tree in Fig. 1A, reconstructed the Ffz1 phylogeny (Additional file 9: Fig. S7), and determined sugar preference for a large fraction of the species (Additional file 5: Fig. S4). In the past, we put forward a hypothesis to explain the patchy distribution of *FFZ1* across the Saccharomycotina which involved two HGT events (from the Pezizomycotina to the W/S clade and from the W/S clade to *Zygosaccharomyces*) [12]. However, the support for the HGT event from the W/S clade to *Zygosaccharomyces* was limited by the low number of genomes available. The updated Ffz1 phylogeny inferred with additional W/S species sequences (Additional file 9: Fig. S7) further supports the two HGT events, as yeast Ffz1 sequences cluster within filamentous fungi, whereas all *Zygosaccharomyces* sequences form a deeply embedded clade within the *Starmerella* subclade. We found again an almost perfect correlation between the presence of the gene and fructophily in the *Starmerella* subclade (subclade 4).

### FFZ1-independent fructophily is patent in a restricted group of Wickerhamiella species

**Fig. 3:**
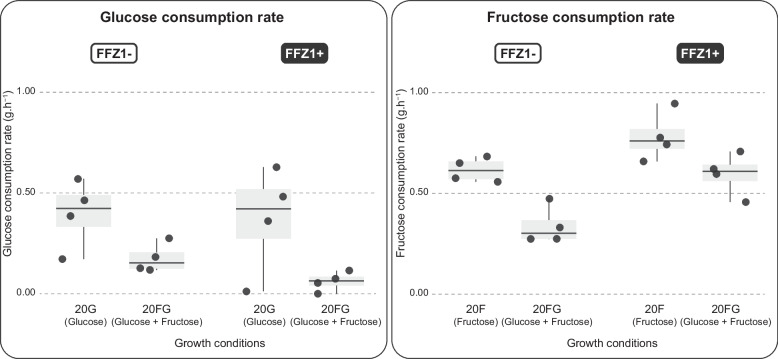
Comparison of glucose and fructose consumption rates in fructophilic species with and without *FFZ1.* Plots denote glucose (left panel) and fructose (right panel) consumption rates in culture media containing either glucose (20G medium) or fructose (20F medium) as sole carbon source or both sugars simultaneously (20FG medium), as indicated below the plots. Within each panel, results for FFZ1 + and FFZ1 − species are shown separately, as indicated, denoting the impact of the presence of the high-capacity fructose transporter on sugar consumption rates under the various conditions. Each datapoint represents a single experiment (replicate experiment can be found in Additional file 6: Table S2 and Additional file 8: Fig. S6B)

In the three *Wickerhamiella* subclades, *FFZ1* was found only in a small number of species, most of which were fructophilic. Surprisingly, however, in subclade 3 (Fig. 1A), fructophily was detected despite the absence of *FFZ1* in seven species. When compared with the sugar consumption profiles observed in *FFZ1*-harboring species (indicated as FFZ1 + in Fig. 1C, e.g., *W. cachassae*), the species lacking the gene are generally slower consumers of fructose (indicated as FFZ1—in Fig. 1C). Also, the sugar consumption profiles in Fig. 1C and Additional file 5: Fig. S4 show that in fructophilic species of the *Wickerhamiella* subclades, both with and without *FFZ1*, glucose tends to be left nearly untouched, which might denote a defect in glucose metabolism. If this were the case, fructophily might be the result of this defect, as opposed to a competition between the two sugars for uptake or phosphorylation. To assess that, we cultivated four fructophilic species lacking *FFZ1* (FFZ1 −) species as well as four fructophilic species harboring *FFZ1* (FFZ1 +) on glucose and fructose separately (using, respectively, 20G and 20F medium) and measured sugar consumption rates under the different conditions (Fig. 3). No defects on glucose utilization were observed in any of the species; even when very small amounts of glucose were consumed, high cell densities were attained reflecting a higher biomass yield on glucose than on fructose (Additional file 6: Table S2). When compared with the individual glucose and fructose consumption rates in medium containing both sugars, rates were generally higher in the single sugar condition (*p*-value = 0.0148, Wilcoxon test) (Fig. 3), showing that there is competition between the utilization of the two sugars as might be expected as a consequence of their very similar metabolisms. Notably, the presence of *FFZ1* appears to slightly mitigate the negative effect of glucose on fructose consumption rates in mixed sugar cultures, while it slightly increases the negative effect of fructose on glucose consumption rates under the same conditions. This competition between the two sugars suggests that they use the same or a largely overlapping set of transporters, with Ffz1 tipping the balance towards fructose uptake when it is present. In line with this, no major differences regarding the number or type of sugar transporters were observed between fructophilic and glucophilic species lacking *FFZ1* (Additional file 10: Table S3), suggesting that the milder fructophily observed in the absence of Ffz1 might be achieved in the absence of an alternative specific fructose transporter. In summary, the fructophilic character of the *FFZ1*-lacking species in subclade 3 suggests either that the available transporters have more affinity for fructose than for glucose or that fructophily in these cases is determined by the kinetic properties of the hexokinases.

### Differences in hexokinase activity do not explain FFZ1-independent fructophily

**Fig. 4:**
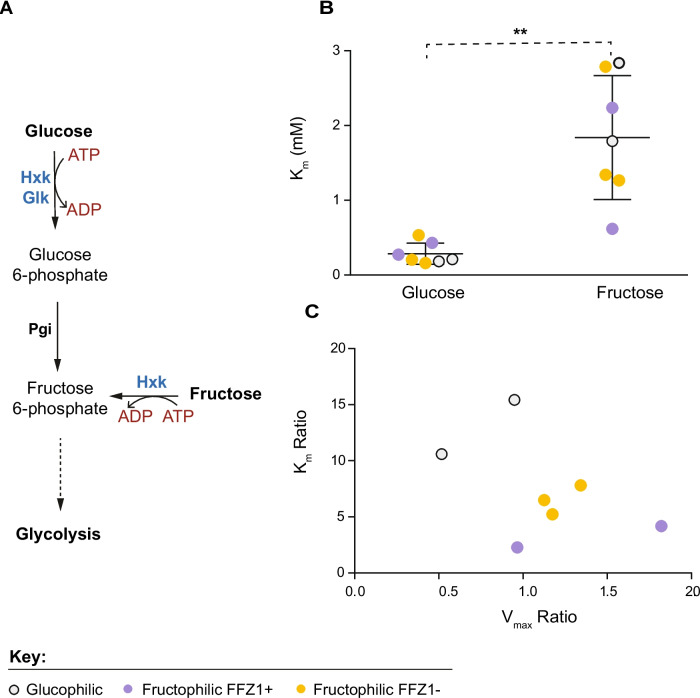
Kinetic properties of hexokinase activity in *Wickerhamiella* species with different trait combinations. **A** First steps of glucose and fructose metabolism after internalization, catalyzed by hexokinase (Hxk), glucokinase (Glk), and phosphoglucoisomerase (Pgi). **B** *K*_m_ of hexokinase activity using either fructose or glucose as substrates, measured in cell-free extracts of the species listed in Additional file 11: Table S4; dots are colored according to species traits, as indicated. *K*_m_ of hexokinase activity for fructose is significantly higher than for glucose (*p*-value = 0.0018, *t*-test). **C** Variation in substrate bias of hexokinase activity as denoted by *K*_m_ (fructose)/*K*_m_ (glucose) ratios for the various species studied, plotted against the respective *V*_max_(fructose)/*V*_max_ (glucose) ratios, showing that hexokinase activity tends to be less unfavorable towards fructose utilization in fructophilic species, color coded as in **B**

As glucose and fructose metabolisms are so similar, the only two metabolic reactions potentially determining a preference for one or the other of the two hexoses are the transport and phosphorylation steps (Fig. 4A). Early in the study of strongly fructophilic *Zygosaccharomyces* species [34], it was established that the transport step was the culprit, later leading to the identification of *FFZ1* [35]. The finding of fructophily independent of *FFZ1* in some *Wickerhamiella* species led us to re-assess whether sugar phosphorylation kinetics might determine a preference for fructose in these species. To that end, glucose and fructose phosphorylation kinetics were determined in cell-free extracts of several *Wickerhamiella* species exhibiting different trait combinations (Fig. 4B; Additional file 11: Table S4), with a focus on sugar preference and the presence/absence of *FFZ1*. The results, shown in Fig. 4B, indicate that a bias of hexokinase activity towards fructose is unlikely to determine fructophily in any of the species examined, as the *K*_m_ for fructose is always considerably higher than the *K*_m_ for glucose. Nevertheless, the various species exhibited markedly different fructose/glucose *K*_m_ and *V*_max_ ratios (Fig. 4C). The two glucophilic species had the lowest *V*_max_ ratios and highest *K*_m_ ratios, which is in line with their preference for glucose, while the fructophilic species tended to have lower *K*_m_ ratios and higher *V*_max_ ratios. This suggests that although sugar phosphorylation does not seem to determine fructophily, hexokinase activity seems to have evolved towards improving fructose metabolism in fructophilic species, both with and without *FFZ1*. Hence, taken together, our results suggest that the transport step is mainly responsible for fructophily in *FFZ1*-lacking species, probably due to the higher affinity for fructose of one or more of the hexose transporters, but that there is also some adjustment of the kinetics of hexokinase to fructophily.

### Higher fructose consumption rates in Wickerhamiella are associated with a metabolic shift towards fermentation

**Fig. 5:**
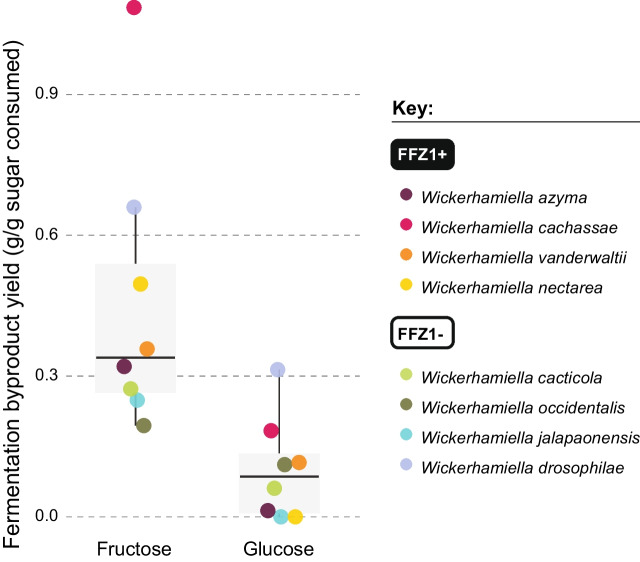
Growth on fructose promotes fermentation. Comparison of global fermentation byproduct formation (ethanol, mannitol, and glycerol together), in the form of global maximum yields, in fructophilic *Wickerhamiella* species possessing or lacking *FFZ1* and cultivated on either glucose (20G medium) or fructose (20F medium) as sole carbon source, as indicated below the plot. Each datapoint represents a single measurement (replicate experiment can be found in Additional file 6: Table S2 and Additional file 8: Fig. S6C)

Higher sugar consumption rates are usually associated with fermentative metabolism. For that reason, we decided to investigate whether the often marked difference between fructose and glucose consumption rates in fructophilic *Wickerhamiella* species (Fig. 3 and Additional file 6: Table S2) reflected a sugar-dependent preference for respiration or fermentation. Notably, even when glucose is left nearly untouched and fructose is efficiently consumed (for instance in *W. nectarea* or *W. jalapaonensis*, Additional file 6: Table S2), the maximum cell density reached on glucose is similar to or higher than that reached on fructose-based medium, suggesting that glucose-grown cells are shifting their metabolism towards the more energy effective respiration at the detriment of fermentation. To ascertain this, we determined the global fermentation byproduct yields (i.e., ethanol, mannitol, and glycerol considered together) for eight fructophilic *Wickerhamiella* species grown separately on glucose and on fructose (Additional file 6: Table S2). In all species, the fermentation byproduct yields were significantly higher on fructose compared to glucose (*p*-value = 0.002742, Wilcoxon Test) (Fig. 5), ranging from 2 × in *W. occidentalis* to 25 × in *W. azyma* (Additional file 6: Table S2). This increase in byproduct formation when fructose is the carbon-source was not restricted to mannitol formation, which might be expected because mannitol can be formed directly from fructose (Additional file 6: Table S2). Instead, in some species, like *W. cachassae*, *W. nectarea*, *W. azyma*, and *W. jalapaonensis*, an increase in glycerol and/or ethanol formation is also observed on fructose relative to glucose based medium. Taken together, these results suggest that in fructophilic *Wickerhamiella* species fructose is more conducive to fermentation than glucose and that this effect seems to be more pronounced for *FFZ1* harboring species.

## Conclusions

### Evolutionary events involving alcoholic fermentation- and fructophily-related genes

**Fig. 6:**
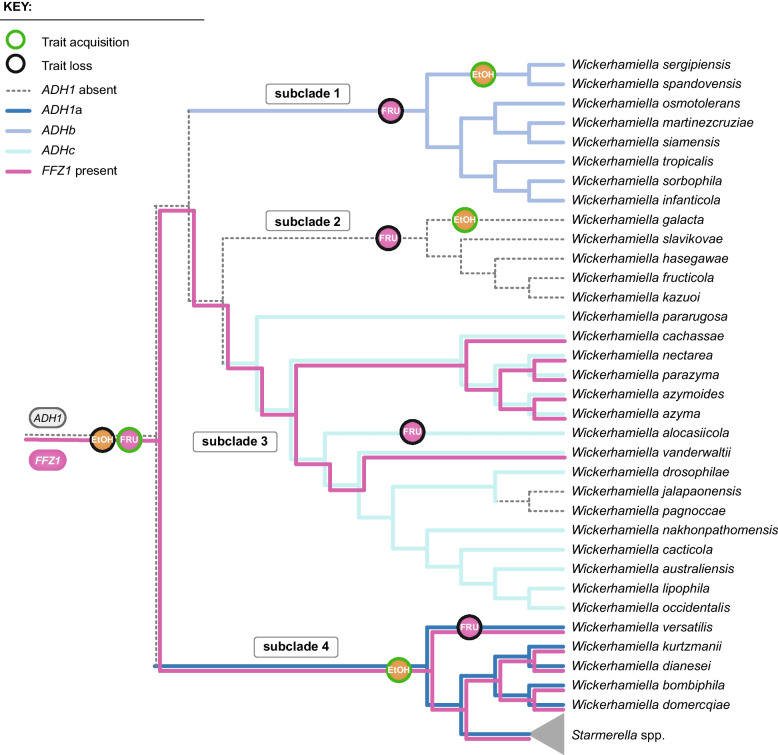
Proposed model representing evolutionary events pertaining to alcoholic fermentation and fructophily in the W/S clade. Horizontal gene transfer and gene loss events involving *FFZ1*, *ADH1a*, *ADH1b*, and *ADH1c* genes were inferred based on the distribution of the genes across the clade and on the phylogenetic analyses of gene trees. The presence/absence of the genes in extant W/S-clade species is depicted by the differently colored tree branches, as indicated. Acquisitions of fructophily (FRU) and alcoholic fermentation (EtOH) are represented close to the relevant tree branches as indicated in the key. Subclades are indicated as defined in Fig. 1A. Traits in *Starmerella* species are generally as indicated, except for a few exceptions shown in Fig. 1A

With the addition of dozens of newly sequenced genomes from species belonging to the W/S clade, either sequenced by us (45, 36 of which sequenced in the course of this work) or retrieved from public databases [18], we robustly reconstructed the set of evolutionary events involving the genes related to alcoholic fermentation (*ADH1*, *ADH6*, *PDC1*, and *ARO10*) and fructophily (*FFZ1*), two traits that have been affected by multiple events of loss and HGT in this clade (Fig. 6).

At least eight HGT events were involved in the (re)acquisition of *ADH*-like and *PDC*-like genes, most likely from distinct bacterial lineages. In the *Starmerella* clade, the acquisition of an Acetobacteraceae Adh1 enzyme perfectly coincides with the reinstatement of robust alcoholic fermentation, while in the *Wickerhamiella* clade, alcoholic fermentation is more patchily distributed, does not correlate with the type of the Adh1 acquired, and takes place even in the absence of obvious candidate genes (i.e., *ADH1* and *ARO10*/*PDC1*). The evidence for multiple HGT events involving these genes, combined with the finding of a well-defined clade where neither Adh1 or Pdc1 of any type were found, provides further support to our previous hypothesis that the entire alcoholic fermentation pathway was lost in the ancestor of the W/S clade and subsequently reacquired multiple times (Fig. 6).

The phylogenetic distribution of *FFZ1* suggests that it was horizontally acquired by the ancestor of the W/S clade and subsequently lost in a few lineages (Fig. 6). Five losses, two involving single species and three encompassing small subclades, suffice to explain the extant phylogenetic distribution of *FFZ1* (Fig. 6). These losses resulted, in three cases, in the concomitant loss of fructophily, while in subclade 3, this trait was maintained in most species despite the loss of *FFZ1* (Fig. 6). Conceivably, this may have happened because (an)other transporter(s) (partly) took over the role of Ffz1. The transporter involved remains to be identified, but its capacity (*V*_max_) may be lower than that of *FFZ1*, given the observed slower fructose consumption rates in these species, when compared with *FFZ1* harboring species. Interestingly, although hexokinase activity denoted higher affinity for glucose than for fructose in all species tested, both glucophilic and fructophilic, the kinetic parameters lean increasingly towards being less unfavorable to fructose consumption in proportion to observed fructose consumption rates.

### A refined hypothesis for the evolution of alcoholic fermentation in the W/S clade

Our survey of the metabolism of fructose and glucose in the W/S clade as described in the previous sections revealed pronounced differences in sugar preference, relative consumption rates, biomass yield, and also in the type and amount of fermentation products formed. On the other hand, all species examined seem to be Crabtree negative, meaning that fermentation takes place as a response to limited oxygen availability (e.g., when high cell densities are attained) rather than to sugar abundance, unlike what is observed, for example, in *S. cerevisiae* [30, 34]*.* As mentioned before, with few exceptions, significant ethanol production is limited to the *Starmerella* subclade. Contrastingly, mannitol, a byproduct rarely produced by yeasts that can be much more readily obtained from fructose than from glucose, is produced by the large majority of W/S-clade species, albeit in highly variable amounts (Fig. 2B and Additional file 6: Table S2). Moreover, we observed in the eight fructophilic *Wickerhamiella* species cultivated separately on glucose and fructose that fructose metabolism is more conducive to the formation of fermentation byproducts (ethanol, mannitol, and glycerol) than glucose metabolism. This does not seem to be solely explained by faster fructose consumption rates because in some cases even when consumption rates were identical or slightly higher for glucose, fermentation byproducts formed only when fructose was the carbon source or were more abundant in the latter case. However, to ascertain whether different regulatory roles of glucose and fructose are also playing a part in the amount and type of fermentative byproducts formed, more rigorous experiments performed in controlled conditions (chemostat) would be required.

Another notable observation is the fact that when cultivated in medium containing high concentrations of both glucose and fructose, the 42 species studied are not divided in cohorts of non-fermentative (*Wickerhamiella* subclades 1–3) and fermentative (*Starmerella*, subclade 4) (Fig. 2A). Instead, collectively, they occupy a wide range from non-fermentative to fermentative metabolic behavior, albeit with a strong tendency for *Wickerhamiella* to populate the lower, less fermentation-prone space characterized by low sugar consumption rates. It is tempting to speculate that such phenotypic plasticity enhanced ecological adaptation and minimized competitive exclusion in nature.

In summary, our observations taken together with the striking parallel evolutionary path of fructophilic bacteria [14, 15] lead us to posit that after the loss of alcoholic fermentation and concomitant with adaptation of the W/S-clade ancestor to the floral niche, the high-capacity fructose transporter *FFZ1* was first horizontally acquired from a Pezizomycotina species. The possibility of a high fructose metabolic flux presumably provided some mitigation of redox imbalance caused by the loss of alcoholic fermentation by offering an alternative way to recycle reduced co-factors in low oxygen conditions through direct conversion of fructose to mannitol (Gonçalves et al. 2019). Mannitol production may in fact be considered a different kind of fermentation in the sense that it impacts redox balance, which could account for its ubiquity in extant W/S-clade species, sometimes as the sole fermentation byproduct. It seems plausible that after this first fermentative pathway was established, the *Starmerella* lineage (subclade 4) remodeled its energy metabolism much further by reinstating alcoholic fermentation, accomplished by resorting to a horizontally acquired bacterial Adh1 and the recruitment of Aro10 to fulfill the role of Pdc. Our evidence [8] suggests that this may have been favorable to the improvement of glucose utilization under fermentative conditions. Our observations indicate that the importance of mannitol formation for redox balance in *Starmerella* may be presently very reduced or inexistent, like in *St. bombicola* [13]. However, as in the latter species that employs mannitol as thermoprotector, other species over the entire clade may have retained mannitol production in relation with physiological needs other than redox balance. Finally, we note that evidence concerning fructose metabolism in the W/S clade suggests that mannitol and ethanol formation are not alternative or complementary pathways but rather form together a metabolic "fermentation node,” possibly also including glycerol formation. However, as observed in *St. bombicola* mutants unable to conduct alcoholic fermentation, glycerol functions in this species as a redox valve compensating the lack of alcoholic fermentation as is often observed in yeasts [8, 36]. Mannitol production collapsed in these mutants along with ethanol synthesis, suggesting the existence of a metabolic or regulatory link between the two reactions that nevertheless seem to fulfill eminently different cellular roles.

In conclusion, the loss of alcoholic fermentation in the W/S-clade ancestor seems to have triggered a bout of unprecedented innovation in the toolkit normally used by yeasts for fermentation, employing a wide range of strategies that seem to go well beyond the utilization of xenologous enzymes. This is best illustrated by the observation of robust alcoholic fermentation in the absence of the respective canonical pathway. It seems likely that the events in the evolutionary history of fermentation and fructophily in the W/S clade set the stage for a radiation that resulted in the emergence of numerous species spanning a widely diverse fermentation landscape with ethanol, mannitol, and glycerol fulfilling different roles, ranging from contributions to redox balance to various forms of stress protection.

## Methods

### Genome sequencing, de novo assembly, and annotation

In the course of this work, the genomes of 36 species in the W/S clade were sequenced (Additional file 1: Table S1). For each species, genomic DNA from overnight grown cultures was isolated as previously described [11]. Paired-end Illumina NextSeq2000 250 bp reads (300 cycles) were obtained at the Genomics Unit of Instituto Gulbenkian Ciência (Oeiras, Portugal). The raw sequenced reads thus obtained and the publicly available reads for other W/S-clade species were then assembled following an in-house pipeline that uses an optimized trimming step (LEADING:3 TRAILING:3 SLIDINGWINDOW:4:15 MINLEN:36) with Trimmomatic v0.39 [37] and SPAdes v3.13.1 [38] for the de novo assembly step. Only contigs with a length over 1000 bp and coverage above 5 × were kept in the final assembly. For low coverage assemblies (< 20x), contigs with a coverage above 1 were kept. Genome quality was assessed for all genomes with QUAST v5.0.2 [39]. For all species, the complete proteome was predicted with AUGUSTUS v 3.3.3 [40] using the complete gene model and *Saccharomyces cerevisiae* as reference.

For the species *W. slavikovae* PYCC 8320, long-read data was also obtained using Oxford Nanopore Technology (ONT), with a MinION flowcell. For de novo assembly, Canu v2.2 [41] was used with default parameters, only adjusting the genome size flag to 10 m. The resulting contigs were corrected with two rounds of Racon v1.5.0 [42], one with the Nanopore reads and the other with Illumina reads. Afterwards, several rounds of Pilon v1.24 [43] run until no changes were seen on the change file were performed using Illumina data. To further increase the contiguity of the assembly, LINKS v1.8.7 [44] was implemented. The resulting assembly was also used as input to predict the proteome using AUGUSTUS v 3.3.3. Sequenced raw reads are openly available at the European Nucleotide Archive (ENA) under the BioProject ID: PRJEB62807 [45]. Genome assemblies are available in figshare (10.6084/m9.figshare.23292737) [46].

### Search for genes relevant for alcoholic fermentation and fructophily

First, HMM profiles for each protein were constructed. For that, Ffz1, Adh1, Adh6, and Pdc1/Aro10 protein sequences from (*W. versatilitis*, *W. domercqiae*, or *St. bombicola*) were used as queries for BLASTp searches in the NCBI refseq database. Hits with an *e*-value lower than 0.001 were retrieved, to a maximum of 100. The sequences recovered for each protein were aligned with MAFFT v7.407 [47] and HMM profiles were constructed in HMMER v3.3.2 [48] (Nov 2020). These HMM profiles were used to score the presence/absence of each gene and the respective copy number using Orthofisher v1.0.5 [49]. Several *e*-value cutoffs were tested starting with the standard parameters (*e*-value cutoff 1e^−3^). After analyzing the results by reciprocal blast, *e*-value 1e^−50^ returned the best results, with no loss of information and few false positives. All results were inspected by blast in the NCBI database and by phylogenetic analysis. Absences were confirmed in the pertinent genome assemblies by tBLASTn.

To determine the sugar transporter gene repertoire across W/S-clade species, we searched for all proteins with sugar transporter signatures by employing an HMM-based search [49] with different degrees of stringency (bitscore: 10, 20, and 80) using the Sugar porter HMM profile as query (Pfam: PF00083, obtained from Pfam-A.hmm using “hmmfetch”) [50].

### Phylogenetic inference

The species phylogeny was reconstructed with single-copy orthogroups (SCO) obtained using Orthofinder v2.5.4 [51] from the predicted W/S-clade proteomes and closest relatives. The SCO were aligned independently using MAFFT v7.407 and then concatenated using a python script (https://github.com/santiagosnchez/ConcatFasta). The concatenated aligned file composed of 273 SCO was then used to infer a maximum likelihood (ML) tree using IQTREE 1.6.11 [52] with a partition flag (-spp), an automatic detection of the best-fitting model of amino acid evolution [53]. Bootstrap support was assessed by 1000 ultrafast bootstrapping replicates [54] and also using the SH-aLRT branch test [55]. Five independent tree searches were conducted (–runs 5), and the tree with the best likelihood score was selected as the one representing the most likely phylogenetic relationships between species. The phylogeny was rooted using *Tortispora caseinolytica* based on the phylogenetic analyses of Shen et al. 2018 [2].

For the phylogenies of Adh1, Adh6, Ffz1, and Pdc1/Aro10, the confirmed sequences retrieved from Orthofisher [49] were added to the datasets published by Gonçalves et al. 2016 [12] and Gonçalves et al. 2018 [8] and aligned with MAFFT v7.407 using an iterative refinement method (E-INS-i). For Adh6, poorly aligned regions were removed with trimal v1.4.rev15 [56] using the “gappyout” option. The aligned datasets were then used to reconstruct a phylogeny with IQTREE, using automatic detection of the best fitting model of evolution and 1000 ultrafast bootstrap replicates. All the phylogenetic trees reconstructed in this work were visualized and edited using iTOL v6 [57].

### Growth assays, sugar and metabolite quantification

For the assessment of fructophily and quantification of fermentation byproducts shown in Fig. 1A and Fig. 2, cultures of 43 W/S-clade species were pre-grown in 10 mL of YP medium (1% (w/v) of yeast extract and 2% (w/v) of peptone) supplemented with 10% (w/v) of fructose and 10% (w/v) of glucose (20FG medium), overnight, at 25 °C with orbital shaking. The pre-grown cultures were inoculated in 30 mL of fresh 20FG medium at an initial optical density (OD_640nm_) of 0.1 and grown under the same conditions. Growth was followed for 150–400 h. At several time points, 1 mL samples were harvested, centrifuged for 5 min at 16,000 × g, and filtered through 0.22-μm nylon filters for high-performance liquid chromatography (HPLC) analysis. Glucose, fructose, ethanol, glycerol, and mannitol were quantified using a carbohydrate analysis column (300 mm by 7.8 mm, Aminex HPX-87P; Bio-Rad) and a differential refractometer (Shodex R-101). The column was kept at 80 °C, and H_2_O was used as the mobile phase at 0.6 mL/min. Data used to calculate sugar consumption rates and byproduct yields shown in Figs. 3 and 5 was similarly obtained except for the utilization of YP medium supplemented with either 20% (w/v) of fructose (20F) or 20% (w/v) of glucose (20G), instead of 20FG medium. For *Wickerhamiella alocasiicola*, only information regarding sugar consumption profiles was obtained, so this species was not included in any of the analyses involving analyses of fermentation byproducts.

### Yields, sugar consumption rates, and fermentation byproduct production rates

All raw data pertaining to the calculation of sugar consumption rates, byproduct production rates, and yields is presented in Additional file 6: Table S2. Rates and yields varied considerably during growth, and the pattern of this variation was in turn quite different among the 42 species. Therefore, for each species, approximate values for rates and yields were calculated using the time point after inoculation for which the highest value was obtained for a certain rate or yield. Specifically, fermentation byproduct production rates (Fig. 2A) were determined each at the time point for which the rate (sum of fermentation byproduct concentrations per unit of time) was the highest. The same time point was used to determine the respective sugar consumption rate. For the correlation between sugar consumption rates and ethanol production rates (Fig. 2A, inset), the same time points were used but only ethanol production was taken into consideration. Similarly, byproduct yields shown in Fig. 2B and Fig. 5 were calculated each at the time point for which the yields (concentration of each byproduct—Fig. 2B—or sum of total fermentation byproduct concentrations—Fig. 5—produced divided by the amount of sugar consumed) was the highest. The global individual sugar consumption rates shown in Fig. 3 were calculated using the total sugar consumed between the point of inoculation and the timepoint after inoculation where sugars were still present at significant concentrations (> 5 g/L). Replicates were performed for all species. The exact values used in each case and all the calculations for both replicates are shown in Additional file 6: Table S2.

### Determination of hexokinase activity

Seven W/S-clade species were pre-grown in 10 mL of 20FG medium overnight, at 25 °C with orbital shaking. The pre-grown cultures were then inoculated in 50 mL of fresh 20FG medium at an initial OD_640nm_ of 0.1 and grown under the same conditions until mid-exponential phase (~ after 24 h of growth). Cells were harvested by centrifugation (4 °C, 10 min at 8421 × g), washed twice with Tris buffer (50 mM triethanolamine hydrochloride and 1 μM PMSF adjust pH 7.6 with NaOH), and concentrated fourfold. The cell pellet was then washed again with Tris buffer, resuspended in 400 μl of lysis buffer (0.1 M triethanolamine hydrochloride, 2 mM MgCl_2_, 1 mM DTT, and 1 μM PMSF), and 200 μL glass beads (212–300 μm) were subsequently added to the cell suspension. The cells were disrupted by six alternating cycles of 1 min vortexing followed by 1 min cooling on ice. Cell debris were removed by centrifugation (4 °C, 20 min at 16,000 × g), and the supernatants were immediately used for enzymatic assays or stored at – 20 °C. Total protein was quantified using the QUBIT Protein Assay (Thermo Fisher Scientific, Waltham, Massachusetts, USA) following the manufacturer’s guidelines. For the hexokinase assay, a master mix solution with the following components was prepared: 50 mM Tris buffer, 10 mM MgCl_2_, 1 mM ATP, 1 mM NADP^+^, 0.2U of glucose-6-P dehydrogenase (Sigma Aldrich) and 1U phosphoglucose isomerase (Sigma Aldrich). Enzymatic assays were performed at 25 °C in 500 μL reaction mixtures containing the abovementioned solution mix and 25 μL of cell-free extract. The reaction was started by adding glucose (0.04 mM, 0.08 mM, 0.16 mM, 0.4 mM, 0.8 mM 1.2 mM, 2 mM, 10 mM, and 50 mM) or fructose (0.05 mM, 0.1 mM, 0.5 mM, 1 mM, 2 mM, 5 mM, 10 mM, 20 mM, and 50 mM) at several concentrations, and reduction of NADP^+^ (formation of NADPH) was monitored spectrophotometrically by an increase in absorbance at 340 nm for 2 min.

### Statistical analysis

Statistical analyses were performed in RStudio with custom scripts under available packages. The Wilcoxon and *t*-test were performed in R using wilcox.test and t.test function (respectively), after testing for the normality of the data with the Shapiro–Wilk normality test (shapiro.test). Phylogenetic correction of the correlations between fermentation byproducts rates and sugar consumption rates (Fig. 2A) was performed using the *pic* (phylogenetic independent contrasts) tool available in the *ape* R library. A pruned phylogeny including all species for which experimental data was obtained was constructed with PhyKIT [58] using the phylogenetic tree in Fig. 1 as input.

### Supplementary Information

Additional file 1: Table S1. List of yeast strains used in this work for the reconstruction of the phylogenomic trees and in experimental assays.

Additional file 2: Fig. S1. Maximum-likelihood phylogeny of Adh1 proteins. (A) The different lineages are represented by different branch colors (red for Saccharomycotina, light brown for other Fungi (i.e., non-Saccharomycotina), orange for the W/S clade and blue for bacteria). Poorly represented lineages (< 10 sequences) are shown in grey. Branches with bootstrap support higher than 95% are indicated by black dots. (B) Pruned maximum-likelihood phylogenies of Adh1 proteins depicting the phylogenetic relationship between the W/S clade and closest related species.

Additional file 3: Fig. S2. Maximum-likelihood phylogeny of Adh6 proteins. (A) The different lineages are represented by different branch colors (red for Saccharomycotina, light brown for other Fungi (i.e., non-Saccharomycotina), orange W/S clade and blue for bacteria). Poorly represented lineages (< 10 sequences) are shown in grey. Branches with bootstrap support higher than 95% are indicated by black dots. (B) Pruned maximum-likelihood phylogenies of Adh6 proteins depicting the phylogenetic relationship between the W/S clade and closest species. Genomic context and position is given for the multiple copies of Adh6d.

Additional file 4: Fig. S3. Maximum-likelihood phylogeny of Aro10 and Pdc1-like proteins. Phylogeny depicting the relationships between W/S-clade Aro10 proteins and their closest relatives in the Saccharomycotina and between *W. versatilis* Pdc1 xenologs and the closest related bacterial pyruvate decarboxylase. Branches with bootstrap support higher than 95% are indicated by black dots. Different lineages are represented by different branch colors (red for Saccharomycotina, light brown for other Fungi (i.e., non-Saccharomycotina), orange W/S clade and blue for bacteria). Clades highlighted in grey (Aro10-like and Pdc1-like) were assigned according to the phylogenetic position of functionally characterized *Saccharomyces cerevisiae* proteins.

Additional file 5: Fig. S4. Consumption profiles across the W/S clade. Quantification of sugar consumption and fermentative products production across 42 species grown in YP medium supplemented with 100 g/L glucose and 100 g/L fructose. Results of two independent experiments for each species are shown.

Additional file 6: Table S2. Raw data for the sugar consumption and fermentative products determined in YP medium supplemented either with 100 g/L glucose, 100 g/L fructose or both. Values (sugar consumption rates, byproduct rates and yields) represented in Figures 2, 3 and 5 are shown for each species and condition.

Additional file 7: Fig. S5. Phylogenetic correction of the correlation between sugar consumption rates and fermentation byproducts production rates across the W/S clade. On the left, original values for sugar consumption rates and fermentation byproducts rates (A) or ethanol production rates (B) are shown. On the left, respective PIC (phylogenetic independent contrasts) corrected values are represented.

Additional file 8: Fig. S6. Results of a replicate experiment concerning correlation of fermentation byproducts rates and sugar consumption rates (A), comparison of glucose and fructose consumption rates in fructophilic species with and without *FFZ1* (B) and comparison of global fermentation byproduct yields in fructophilic *Wickerhamiella* species cultivated on either glucose or fructose (C).

Additional file 9: Fig. S7. Maximum-likelihood phylogeny of the Ffz1 transporter. Phylogeny depicting the relationships between W/S clade Ffz1 proteins and their closest relatives. The different lineages are represented by different branch colors (red for Zygosaccharomyces spp., light orange for other Pezizomycotina and blue for W/S clade) as indicated in the key. Bootstrap support values are indicated below the respective branch.

Additional file 10: Table S3. Sugar porter HMM-based search in *Wickerhamiella* species. The hits obtained for each of the bitscore threshold values (10, 20 and 80) are presented. A BLASTp search against the NCBI database was performed for *Wickerhamiella* *cacticola* using the three thresholds to evaluate the number of false positives and false negatives. The threshold that yielded the best results was 20. The best BLASTp hits for relevant *Wickerhamiella* species (glucophilic without *FFZ1* and fructophilic with and without *FFZ1*) are indicated.

Additional file 11: Table S4. Kinetic parameters of hexokinase activity for seven W/S clade species with different trait combinations. No competitive experiments were performed, which might reveal additional properties of hexokinases in the various species.

## Data Availability

All data generated or analyzed during this study are included in this published article and its supplementary files. Genome assemblies produced in this work have been deposited on figshare (10.6084/m9.figshare.23292737) [46]. Sequenced raw reads are openly available at the European Nucleotide Archive (ENA) under the BioProject ID: PRJEB62807  [45].

## Abbreviations

W/S clade: *Wickerhamiella*/*Starmerella* clade
HGT: Horizontal gene transfer
*St*.: *Starmerella*
*W.*: *Wickerhamiella*
*S. cerevisiae*: *Saccharomyces cerevisiae*
MRCA: Most recent common ancestor
SCO: Single-copy orthogroups
20G: Yeast peptone supplemented with 20% glucose medium
20F: Yeast peptone supplemented with 20% fructose medium
20FG: Yeast peptone supplemented with 10% glucose and 10% fructose medium
K_m_: Michaelis constant
V_max_: Maximum velocity
PMSF: Phenylmethylsulfonyl fluoride

## References

[1] Li Y, Steenwyk JL, Chang Y, Wang Y, James TY, Stajich JE (2021). A genome-scale phylogeny of the kingdom Fungi. Curr Biol.

[2] Shen XX, Opulente DA, Kominek J, Zhou X, Steenwyk JL, Buh KV (2018). Tempo and mode of genome evolution in the budding yeast subphylum. Cell.

[3] Opulente DA, Rollinson EJ, Bernick-Roehr C, Hulfachor AB, Rokas A, Kurtzman CP (2018). Factors driving metabolic diversity in the budding yeast subphylum. BMC Biol.

[4] Opulente DA, Abigail Leavitt L, Marie-Claire H, John FW, Chao L, Yonglin L, et al. Genomic and ecological factors shaping specialism and generalism across an entire subphylum. bioRxiv. 2023:2023.06.19.545611.

[5] Riley R, Haridas S, Wolfe KH, Lopes MR, Hittinger CT, Goker M (2016). Comparative genomics of biotechnologically important yeasts. Proc Natl Acad Sci USA.

[6] Steenwyk JL, Opulente DA, Kominek J, Shen XX, Zhou X, Labella AL (2019). Extensive loss of cell-cycle and DNA repair genes in an ancient lineage of bipolar budding yeasts. PLoS Biol.

[7] Gonçalves P, Gonçalves C, Brito PH, Sampaio JP (2020). The Wickerhamiella/Starmerella clade—a treasure trove for the study of the evolution of yeast metabolism. Yeast (Chichester, England).

[8] Gonçalves C, Wisecaver JH, Kominek J, Oom MS, Leandro MJ, Shen XX, et al. Evidence for loss and reacquisition of alcoholic fermentation in a fructophilic yeast lineage. Elife. 2018;7:e33034. 10.7554/eLife.33034 PMC5897096 29648535

[9] Kominek J, Doering DT, Opulente DA, Shen XX, Zhou X, DeVirgilio J (2019). Eukaryotic acquisition of a bacterial operon. Cell.

[10] Gonçalves C, Gonçalves P (2019). Multilayered horizontal operon transfers from bacteria reconstruct a thiamine salvage pathway in yeasts. Proc Natl Acad Sci USA.

[11] Gonçalves C, Marques M, Gonçalves P (2022). Contrasting strategies for sucrose utilization in a floral yeast clade. mSphere..

[12] Gonçalves C, Coelho MA, Salema-Oom M, Gonçalves P (2016). Stepwise functional evolution in a fungal sugar transporter family. Mol Biol Evol.

[13] Gonçalves C, Ferreira C, Gonçalves LG, Turner DL, Leandro MJ, Salema-Oom M (2019). A new pathway for mannitol metabolism in yeasts suggests a link to the evolution of alcoholic fermentation. Front Microbiol.

[14] Endo A, Maeno S, Tanizawa Y, Kneifel W, Arita M, Dicks L, et al. Fructophilic lactic acid bacteria, a unique group of fructose-fermenting microbes. Appl Environ Microbiol. 2018;84(19):e01290–18. 10.1128/AEM.01290-18 PMC6146980 30054367

[15] Endo A, Tanaka N, Oikawa Y, Okada S, Dicks L (2014). Fructophilic characteristics of Fructobacillus spp. may be due to the absence of an alcohol/acetaldehyde dehydrogenase gene (adhE). Curr Microbiol..

[16] Filannino P, Di Cagno R, Tlais AZA, Cantatore V, Gobbetti M (2019). Fructose-rich niches traced the evolution of lactic acid bacteria toward fructophilic species. Crit Rev Microbiol.

[17] Endo A, Maeno S, Tanizawa Y, Kneifel W, Arita M, Dicks L (2018). Fructophilic lactic acid bacteria, a unique group of fructose-fermenting microbes. Appl Environ Microbiol.

[18] Endo A, Tanizawa Y, Tanaka N, Maeno S, Kumar H, Shiwa Y (2015). Comparative genomics of Fructobacillus spp. and Leuconostoc spp. reveals niche-specific evolution of Fructobacillus spp. BMC genomics..

[19] Maeno S, Kajikawa A, Dicks L, Endo A (2019). Introduction of bifunctional alcohol/acetaldehyde dehydrogenase gene (adhE) in F*ructobacillus fructosus* settled its fructophilic characteristics. Res Microbiol.

[20] Maeno S, Tanizawa Y, Kanesaki Y, Kubota E, Kumar H, Dicks L (2016). Genomic characterization of a fructophilic bee symbiont *Lactobacillus kunkeei* reveals its niche-specific adaptation. Syst Appl Microbiol.

[21] Sanderson MJ, McMahon MM, Steel M (2010). Phylogenomics with incomplete taxon coverage: the limits to inference. BMC Evol Biol.

[22] de Smidt O, du Preez JC, Albertyn J (2008). The alcohol dehydrogenases of *Saccharomyces cerevisiae*: a comprehensive review. FEMS Yeast Res.

[23] Liang J-J, Zhang M-L, Ding M, Mai Z-M, Wu S-X, Du Y (2014). Alcohol dehydrogenases from Kluyveromyces marxianus: heterologous expression in *Escherichia coli* and biochemical characterization. BMC Biotechnol..

[24] Karaoğlan M, Erden-Karaoğlan F, Yılmaz S, İnan M (2020). Identification of major *ADH* genes in ethanol metabolism of *Pichia pastoris*. Yeast (Chichester, England).

[25] Larroy C, Rosario Fernandez M, Gonzalez E, Pares X, Biosca JA (2003). Properties and functional significance of *Saccharomyces cerevisiae* ADHVI. Chem Biol Interact.

[26] Larroy C, Fernández MR, González E, Parés X, Biosca JA (2002). Characterization of the *Saccharomyces cerevisiae* YMR318C (*ADH6*) gene product as a broad specificity NADPH-dependent alcohol dehydrogenase: relevance in aldehyde reduction. Biochem J.

[27] Russell DW, Smith M, Williamson VM, Young ET (1983). Nucleotide sequence of the yeast alcohol dehydrogenase II gene. J Biol Chem.

[28] Kessler D, Leibrecht I, Knappe J (1991). Pyruvate-formate-lyase-deactivase and acetyl-CoA reductase activities of *Escherichia coli* reside on a polymeric protein particle encoded by adhE. FEBS Lett.

[29] Wang H, Li Q, Kuang X, Xiao D, Han X, Hu X (2018). Functions of aldehyde reductases from Saccharomyces cerevisiae in detoxification of aldehyde inhibitors and their biotechnological applications. Appl Microbiol Biotechnol.

[30] Liu ZL (2018). Understanding the tolerance of the industrial yeast *Saccharomyces cerevisiae* against a major class of toxic aldehyde compounds. Appl Microbiol Biotechnol.

[31] Yang D-D, Billerbeck GMd, Zhang J-J, Rosenzweig F, Francois J-M (2018). Deciphering the origin, evolution, and physiological function of the subtelomeric aryl-alcohol dehydrogenase gene family in the yeast Saccharomyces cerevisiae. Appl Environ Microbiol..

[32] Pfeiffer T, Morley A (2014). An evolutionary perspective on the Crabtree effect. Front Mol Biosci..

[33] Cabral S, Prista C, Loureiro-Dias MC, Leandro MJ (2015). Occurrence of FFZ genes in yeasts and correlation with fructophilic behaviour. Microbiology (Reading, England).

[34] Sousa-Dias S, Gonçalves T, Leyva JS, Peinado JM, Loureiro-Dias MC (1996). Kinetics and regulation of fructose and glucose transport systems are responsible for fructophily in *Zygosaccharomyces bailii*. Microbiology (Reading, England).

[35] Pina C, Goncalves P, Prista C, Loureiro-Dias MC (2004). Ffz1, a new transporter specific for fructose from *Zygosaccharomyces bailii*. Microbiology (Reading, England).

[36] de Smidt O, du Preez JC, Albertyn J (2012). Molecular and physiological aspects of alcohol dehydrogenases in the ethanol metabolism of *Saccharomyces cerevisiae*. FEMS Yeast Res.

[37] Bolger AM, Lohse M, Usadel B (2014). Trimmomatic: a flexible trimmer for Illumina sequence data. Bioinformatics (Oxford, England).

[38] Bankevich A, Nurk S, Antipov D, Gurevich AA, Dvorkin M, Kulikov AS (2012). SPAdes: a new genome assembly algorithm and its applications to single-cell sequencing. J Comput Biol.

[39] Gurevich A, Saveliev V, Vyahhi N, Tesler G (2013). QUAST: quality assessment tool for genome assemblies. Bioinformatics (Oxford, England).

[40] Stanke M, Keller O, Gunduz I, Hayes A, Waack S, Morgenstern B (2006). AUGUSTUS: ab initio prediction of alternative transcripts. Nucleic Acids Res..

[41] Koren S, Walenz BP, Berlin K, Miller JR, Bergman NH, Phillippy AM (2017). Canu: scalable and accurate long-read assembly via adaptive k-mer weighting and repeat separation. Genome Res.

[42] Vaser R, Sović I, Nagarajan N, Šikić M (2017). Fast and accurate de novo genome assembly from long uncorrected reads. Genome Res.

[43] Walker BJ, Abeel T, Shea T, Priest M, Abouelliel A, Sakthikumar S (2014). Pilon: an integrated tool for comprehensive microbial variant detection and genome assembly improvement. PLoS ONE.

[44] Warren RL, Yang C, Vandervalk BP, Behsaz B, Lagman A, Jones SJM (2015). LINKS: scalable, alignment-free scaffolding of draft genomes with long reads. GigaScience.

[45] Pontes A, Paraíso F, Silva M, Lagoas C, Aires A, Brito PH, et al. Extensive remodeling of sugar metabolism through gene loss and horizontal gene transfer in a eukaryotic lineage. European Nucleotide Archive (ENA) PRJEB62807. 2024. 10.1186/s12915-024-01929-7 PMC11140947 38816863

[46] Extensive remodeling of sugar metabolism through gene loss and horizontal gene transfer in a eukaryotic lineage. Online resource. Figshare. 10.6084/m9.figshare.23292737.v1 [Internet]. 2024. 10.1186/s12915-024-01929-7 PMC11140947 38816863

[47] Katoh K, Standley DM (2014). MAFFT: iterative refinement and additional methods. Methods Mol Biol (Clifton, NJ).

[48] Finn RD, Clements J, Eddy SR (2011). HMMER web server: interactive sequence similarity searching. Nucleic Acids Res..

[49] Steenwyk JL, Rokas A (2021). Orthofisher: a broadly applicable tool for automated gene identification and retrieval. G3 Genes Genomes Genetics..

[50] Finn RD, Bateman A, Clements J, Coggill P, Eberhardt RY, Eddy SR (2014). Pfam: the protein families database. Nucleic Acids Res..

[51] Emms DM, Kelly S (2019). OrthoFinder: phylogenetic orthology inference for comparative genomics. Genome Biol.

[52] Nguyen LT, Schmidt HA, von Haeseler A, Minh BQ (2015). IQ-TREE: a fast and effective stochastic algorithm for estimating maximum-likelihood phylogenies. Mol Biol Evol.

[53] Kalyaanamoorthy S, Minh BQ, Wong TKF, von Haeseler A, Jermiin LS (2017). ModelFinder: fast model selection for accurate phylogenetic estimates. Nat Methods.

[54] Hoang DT, Chernomor O, von Haeseler A, Minh BQ, Vinh LS (2018). UFBoot2: improving the ultrafast bootstrap approximation. Mol Biol Evol.

[55] Guindon S, Dufayard J-F, Lefort V, Anisimova M, Hordijk W, Gascuel O (2010). New algorithms and methods to estimate maximum-likelihood phylogenies: assessing the performance of PhyML 3.0. Systematic biology..

[56] Capella-Gutierrez S, Silla-Martinez JM, Gabaldon T (2009). trimAl: a tool for automated alignment trimming in large-scale phylogenetic analyses. Bioinformatics (Oxford, England).

[57] Letunic I, Bork P (2021). Interactive Tree Of Life (iTOL) v5: an online tool for phylogenetic tree display and annotation. Nucleic Acids Res.

[58] Steenwyk JL, Buida TJ, Labella AL, Li Y, Shen XX, Rokas A (2021). PhyKIT: a broadly applicable UNIX shell toolkit for processing and analyzing phylogenomic data. Bioinformatics (Oxford, England).

