# Supplementary material for: Extensive remodeling of sugar metabolism through gene loss and horizontal gene transfer in a eukaryotic lineage
Source: BMC Biol. 2024 May 30;22:128. doi: 10.1186/s12915-024-01929-7 (PMC11140947; doi:10.1186/s12915-024-01929-7)
Supplement: Supplementary file 2 — Additional file 2: Fig. S1. Maximum-likelihood phylogeny of Adh1 proteins. (A) The different lineages are represented by different branch colors (red for Saccharomycotina, light brown for other Fungi (i.e., non-Saccharomycotina), orange for the W/S clade and blue for bacteria). Poorly represented lineages (< 10 sequences) are shown in grey. Branches with bootstrap support higher than 95% are indicated by black dots. (B) Pruned maximum-likelihood phylogenies of Adh1 proteins depicting the phylogenetic relationship between the W/S clade and closest related species. [file 12915_2024_1929_MOESM2_ESM.pdf]

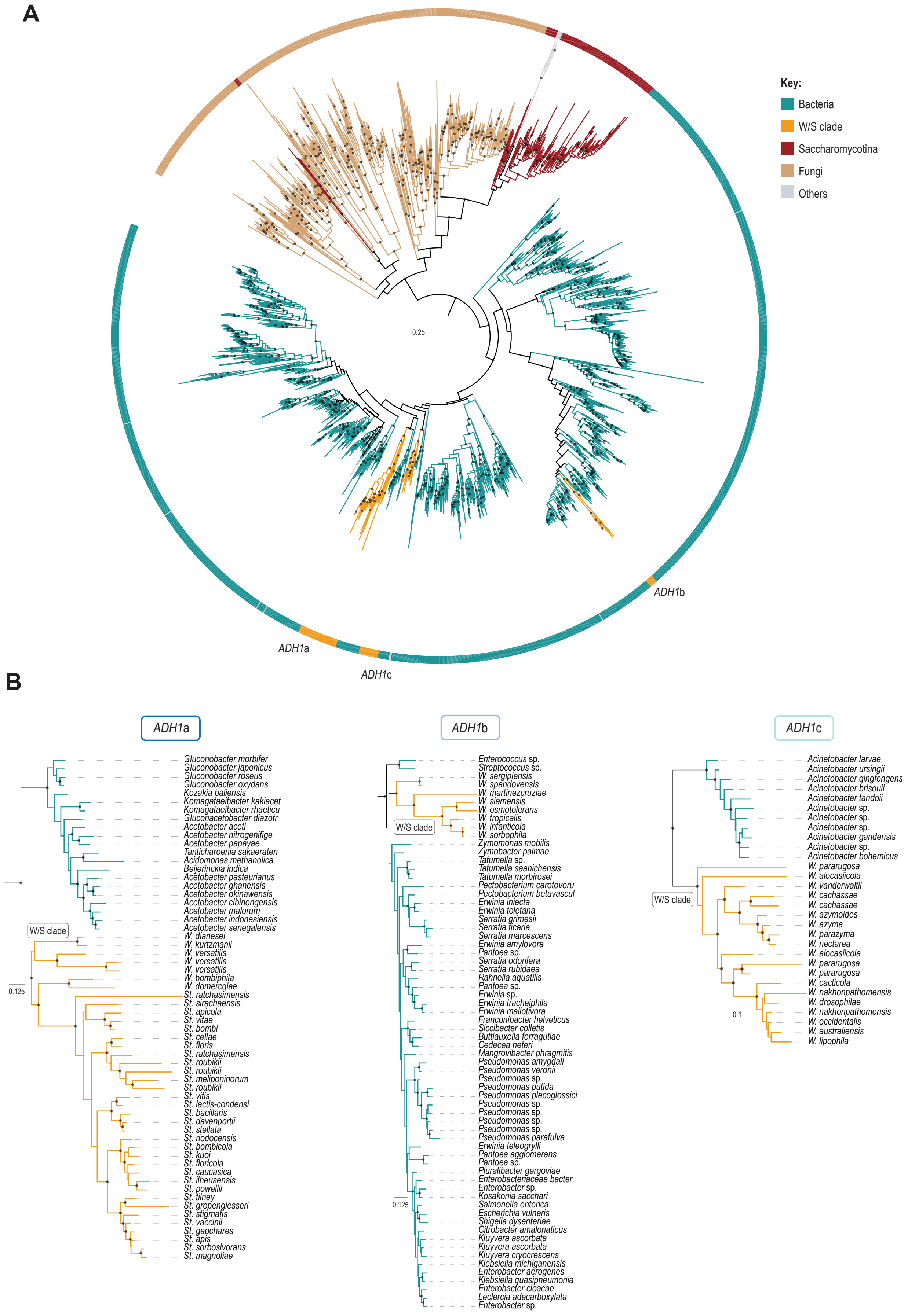

Supplementary Figure S1. Fig. S1. Maximum-likelihood phylogeny of Adh1 proteins. (A) The different lineages are represented by different branch colors (red for Saccharomycotina, light brown for other Fungi (i.e., non-Saccharomycotina), orange for the W/S clade and blue for bacteria). Poorly represented lineages (< 10 sequences) are shown in grey. Branches with bootstrap support higher than 95% are indicated by black dots. (B) Pruned maximum-likelihood phylogenies of Adh1 proteins depicting the phylogenetic relationship between the W/S clade and closest related species.
