# Supplementary material for: Extensive remodeling of sugar metabolism through gene loss and horizontal gene transfer in a eukaryotic lineage
Source: BMC Biol. 2024 May 30;22:128. doi: 10.1186/s12915-024-01929-7 (PMC11140947; doi:10.1186/s12915-024-01929-7)
Supplement: Supplementary file 4 — Additional file 4: Fig. S3. Maximum-likelihood phylogeny of Aro10 and Pdc1-like proteins. Phylogeny depicting the relationships between W/S-clade Aro10 proteins and their closest relatives in the Saccharomycotina and between W. versatilis Pdc1 xenologs and the closest related bacterial pyruvate decarboxylase. Branches with bootstrap support higher than 95% are indicated by black dots. Different lineages are represented by different branch colors (red for Saccharomycotina, light brown for other Fungi (i.e., non-Saccharomycotina), orange W/S clade and blue for bacteria). Clades highlighted in grey (Aro10-like and Pdc1-like) were assigned according to the phylogenetic position of functionally characterized Saccharomyces cerevisiae proteins. [file 12915_2024_1929_MOESM4_ESM.pdf]

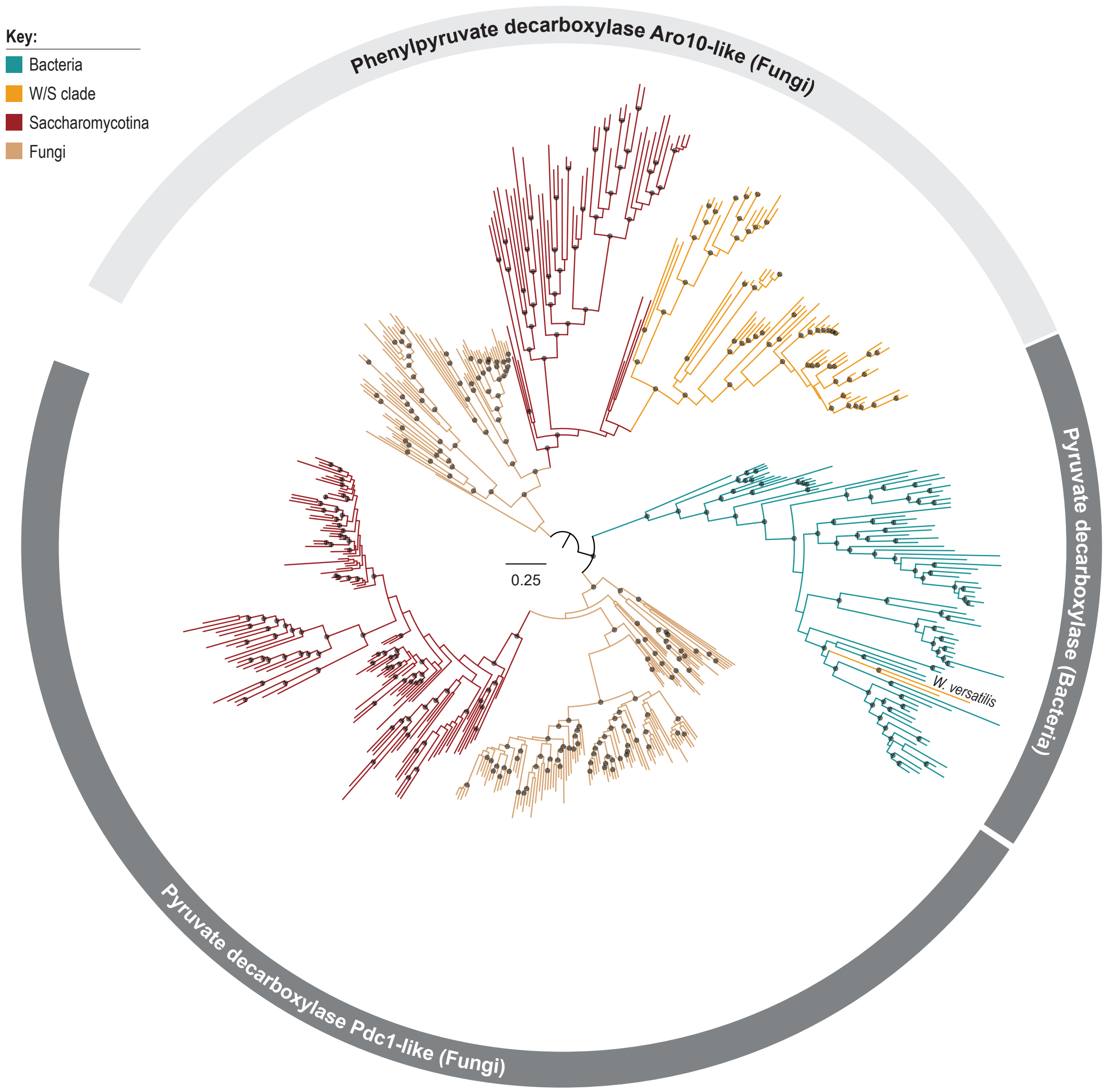

Supplementary Figure S3. Maximum-likelihood phylogeny of Aro10 and Pdc1-like proteins. Phylogeny depicting the relationships between W/S clade Aro10 proteins and their closest relatives in the Saccharomycotina and between *W. versatilis* Pdc1 xenologs and the closest related bacterial pyruvate decarboxylase. Branches with bootstrap support higher than 95% are indicated by black dots. Different lineages are represented by different branch colors (red for Saccharomycotina, light brown for other Fungi (i.e., non-Saccharomycotina), orange W/S clade and blue for bacteria). Clades highlighted in grey (Aro10-like and Pdc1-like) were assigned according to the phylogenetic position of functionally characterized *Saccharomyces cerevisiae* proteins.
