# Supplementary material for: Extensive remodeling of sugar metabolism through gene loss and horizontal gene transfer in a eukaryotic lineage
Source: BMC Biol. 2024 May 30;22:128. doi: 10.1186/s12915-024-01929-7 (PMC11140947; doi:10.1186/s12915-024-01929-7)
Supplement: Supplementary file 5 — Additional file 5: Fig. S4. Consumption profiles across the W/S clade. Quantification of sugar consumption and fermentative products production across 42 species grown in YP medium supplemented with 100 g/L glucose and 100 g/L fructose. Results of two independent experiments for each species are shown. [file 12915_2024_1929_MOESM5_ESM.pdf]

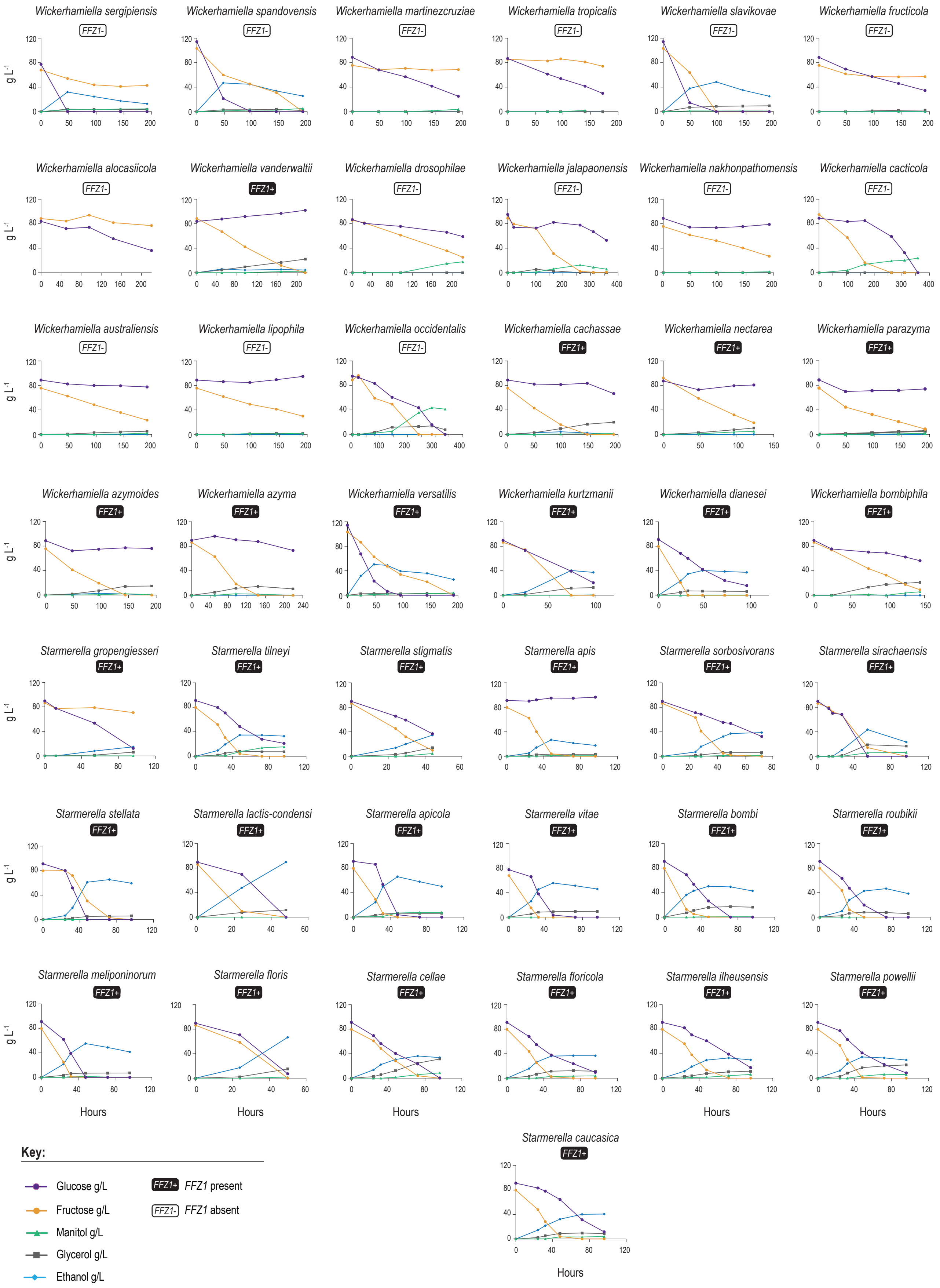

Wickerhamiella sergipiensis FFZ1-
Wickerhamiella spandovensis FFZ1-
Wickerhamiella martinezcruziae FFZ1-
Wickerhamiella tropicalis FFZ1-
Wickerhamiella slavikovae FFZ1-
Wickerhamiella fructicola FFZ1-
Wickerhamiella alocasiicola FFZ1-
Wickerhamiella vanderwaltii FFZ1+
Wickerhamiella drosophilae FFZ1-
Wickerhamiella jalapaonensis FFZ1-
Wickerhamiella nakhonpathomensis FFZ1-
Wickerhamiella cacticola FFZ1-
Wickerhamiella australiensis FFZ1-
Wickerhamiella lipophila FFZ1-
Wickerhamiella occidentalis FFZ1-
Wickerhamiella cachassae FFZ1+
Wickerhamiella nectarea FFZ1+
Wickerhamiella parazyma FFZ1+
Wickerhamiella azymoides FFZ1+
Wickerhamiella azyma FFZ1+
Wickerhamiella versatilis FFZ1+
Wickerhamiella kurtzmanii FFZ1+
Wickerhamiella dianesei FFZ1+
Wickerhamiella bombiphila FFZ1+
Starmerella gropengiesseri FFZ1+
Starmerella tilneyi FFZ1+
Starmerella stigmatis FFZ1+
Starmerella apis FFZ1+
Starmerella sorbosivorans FFZ1+
Starmerella sirachaensis FFZ1+
Starmerella stellata FFZ1+
Starmerella lactis-condensi FFZ1+
Starmerella apicola FFZ1+
Starmerella vitae FFZ1+
Starmerella bombi FFZ1+
Starmerella roubikii FFZ1+
Starmerella meliponinorum FFZ1+
Starmerella floris FFZ1+
Starmerella cellae FFZ1+
Starmerella floricola FFZ1+
Starmerella ilheusensis FFZ1+
Starmerella powellii FFZ1+
Starmerella caucasica FFZ1+
g L-1
Hours
Key:
Glucose g/L
Fructose g/L
Manitol g/L
Glycerol g/L
Ethanol g/L
FFZ1+ FFZ1 present
FFZ1- FFZ1 absent

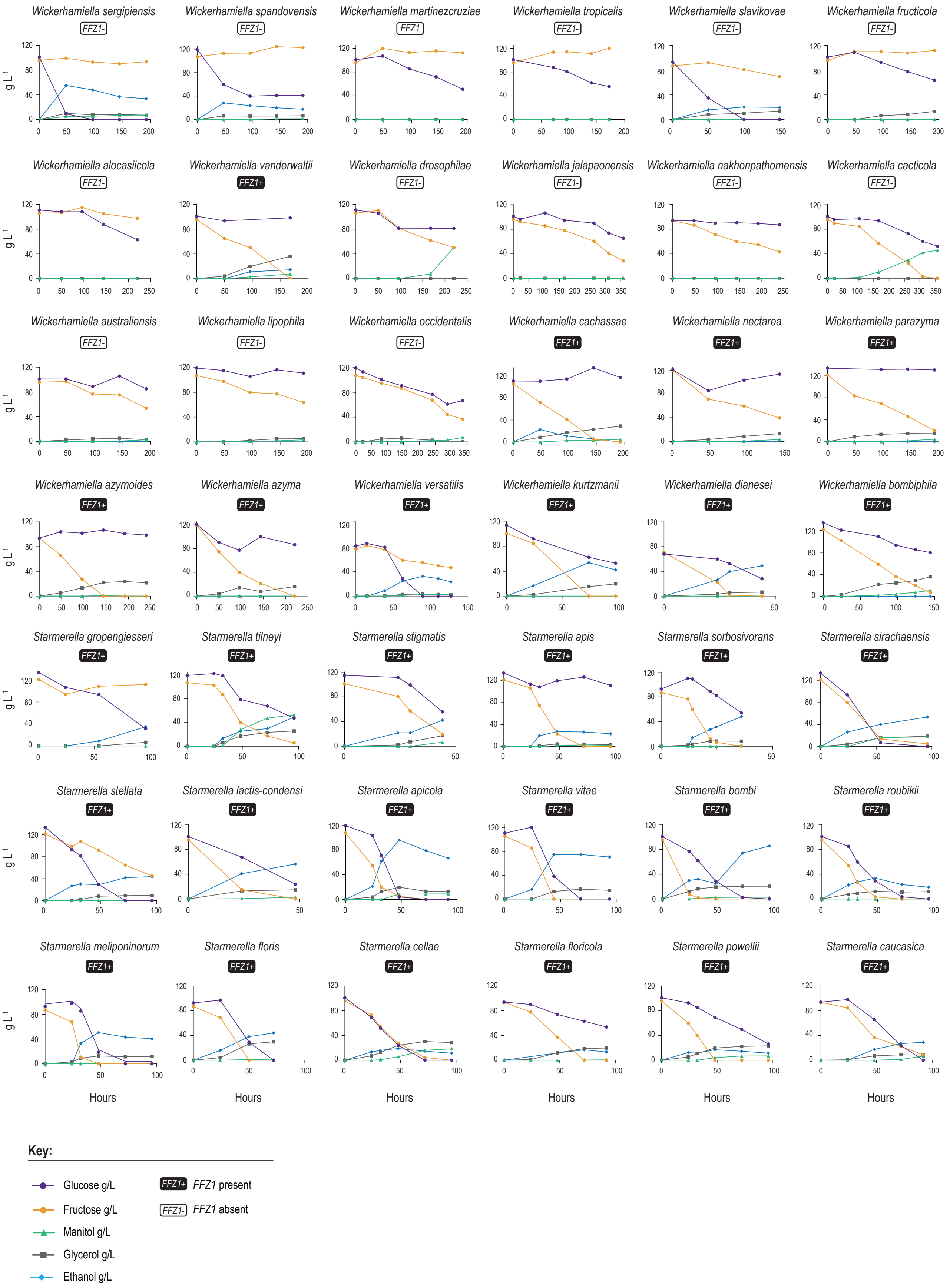

Supplementary Figure S4. Consumption profiles across the W/S clade. Quantification of sugar consumption and fermentative products production across 42 species grown in YP medium supplemented with 100 g/L glucose and 100 g/L fructose. Results of two independent experiments for each species are shown.
