# Supplementary material for: Extensive remodeling of sugar metabolism through gene loss and horizontal gene transfer in a eukaryotic lineage
Source: BMC Biol. 2024 May 30;22:128. doi: 10.1186/s12915-024-01929-7 (PMC11140947; doi:10.1186/s12915-024-01929-7)
Supplement: Supplementary file 7 — Additional file 7: Fig. S5. Phylogenetic correction of the correlation between sugar consumption rates and fermentation byproducts production rates across the W/S clade. On the left, original values for sugar consumption rates and fermentation byproducts rates (A) or ethanol production rates (B) are shown. On the left, respective PIC (phylogenetic independent contrasts) corrected values are represented. [file 12915_2024_1929_MOESM7_ESM.pdf]

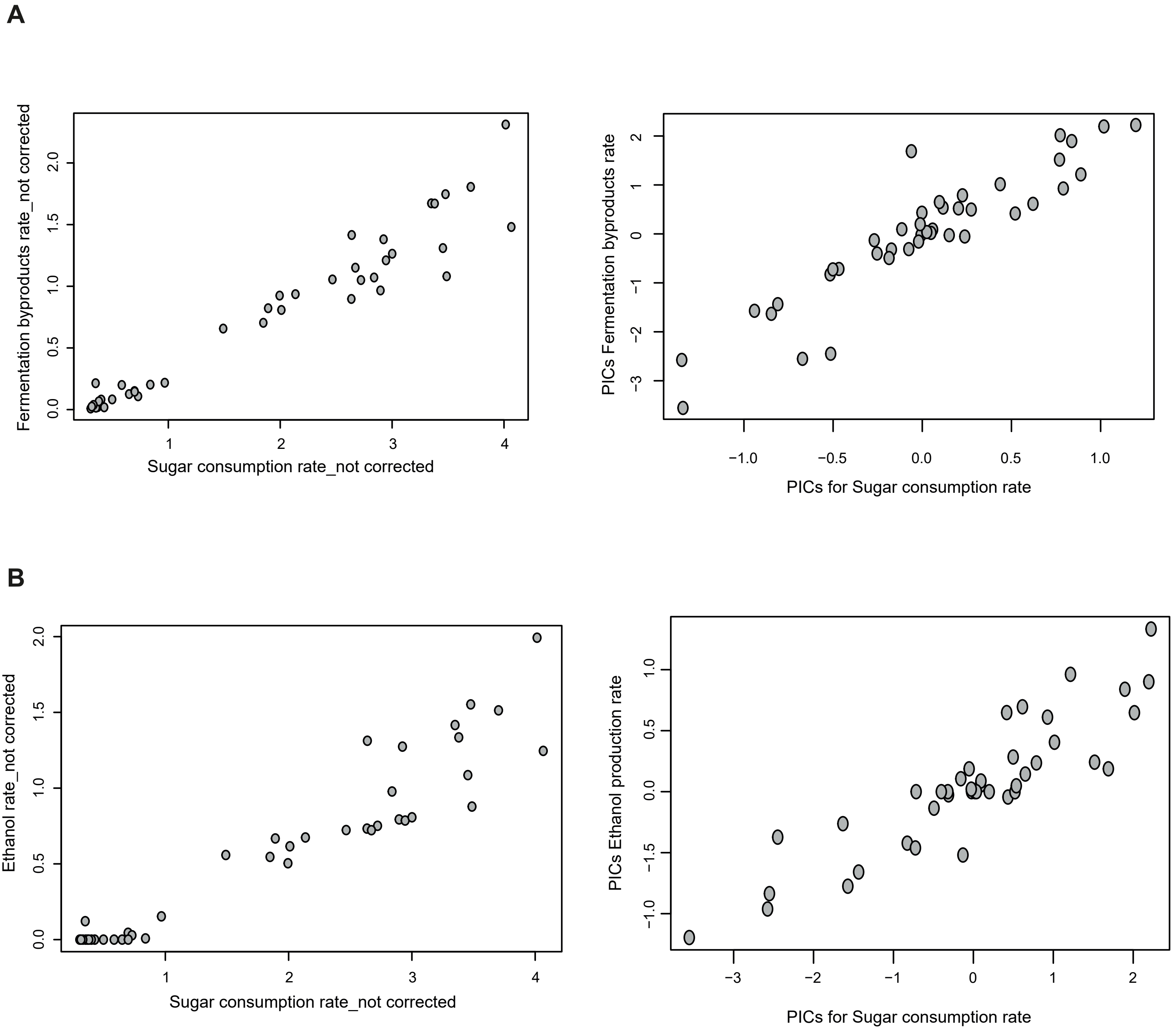

Supplementary Figure S5. Phylogenetic correction of the correlation between sugar consumption rates and fermentation byproducts production rates across the W/S clade. On the left, original values for sugar consumption rates and fermentation byproducts rates (A) or ethanol production rates (B) are shown. On the left, respective PIC (phylogenetic independent contrasts) corrected values are represented.
