# Supplementary material for: Extensive remodeling of sugar metabolism through gene loss and horizontal gene transfer in a eukaryotic lineage
Source: BMC Biol. 2024 May 30;22:128. doi: 10.1186/s12915-024-01929-7 (PMC11140947; doi:10.1186/s12915-024-01929-7)
Supplement: Supplementary file 8 — Additional file 8: Fig. S6. Results of a replicate experiment concerning correlation of fermentation byproducts rates and sugar consumption rates (A), comparison of glucose and fructose consumption rates in fructophilic species with and without FFZ1 (B) and comparison of global fermentation byproduct yields in fructophilic Wickerhamiella species cultivated on either glucose or fructose (C). [file 12915_2024_1929_MOESM8_ESM.pdf]

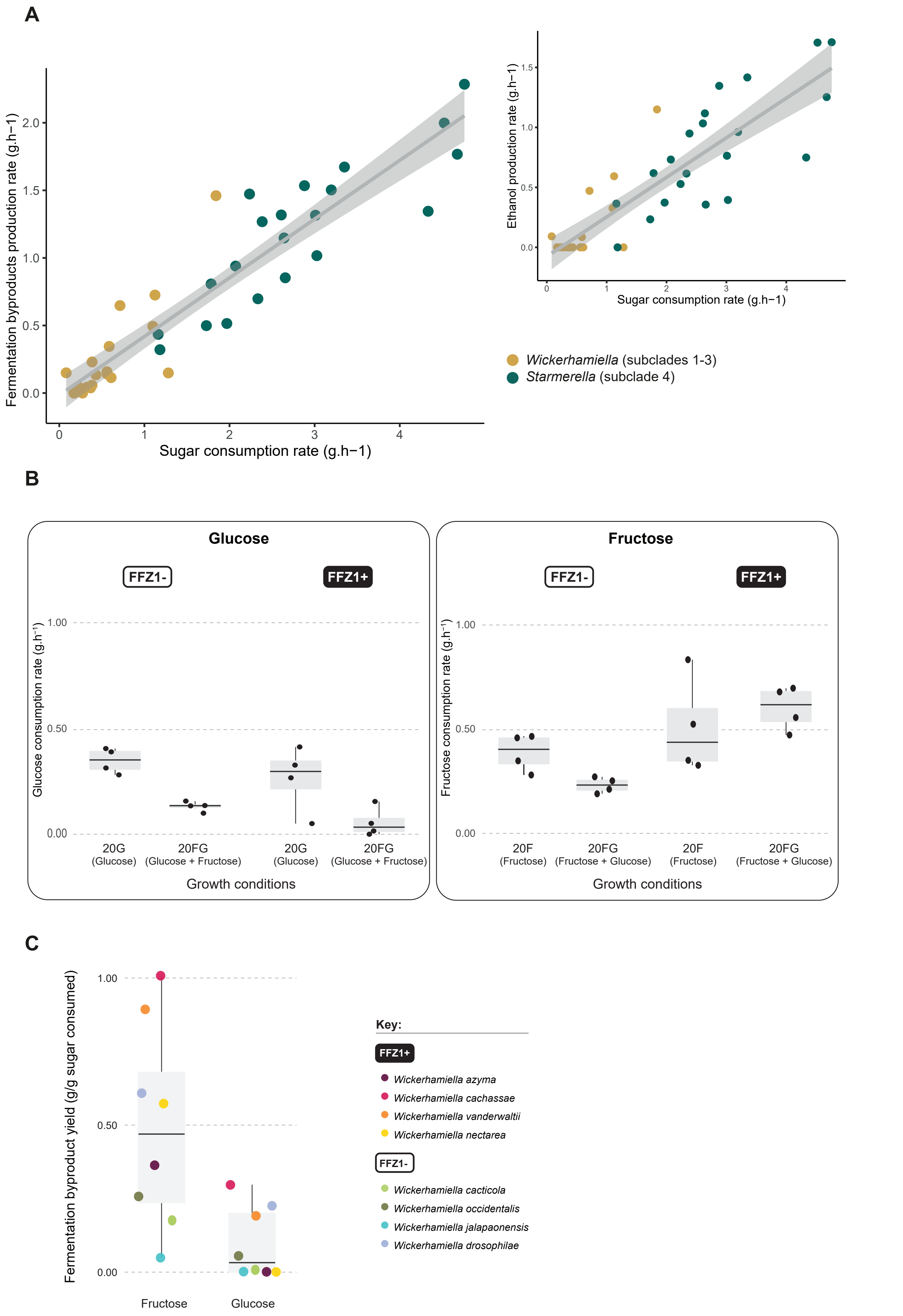

Supplementary Figure S6. Results of a replicate experiment concerning correlation of fermentation byproducts rates and sugar consumption rates (A), comparison of glucose and fructose consumption rates in fructophilic species with and without FFZ1 (B) and comparison of global fermentation byproduct yields in fructophilic Wickerhamiella species cultivated on either glucose or fructose (C).
