# Supplementary material for: Extensive remodeling of sugar metabolism through gene loss and horizontal gene transfer in a eukaryotic lineage
Source: BMC Biol. 2024 May 30;22:128. doi: 10.1186/s12915-024-01929-7 (PMC11140947; doi:10.1186/s12915-024-01929-7)
Supplement: Supplementary file 9 — Additional file 9: Fig. S7. Maximum-likelihood phylogeny of the Ffz1 transporter. Phylogeny depicting the relationships between W/S clade Ffz1 proteins and their closest relatives. The different lineages are represented by different branch colors (red for Zygosaccharomyces spp., light orange for other Pezizomycotina and blue for W/S clade) as indicated in the key. Bootstrap support values are indicated below the respective branch. [file 12915_2024_1929_MOESM9_ESM.pdf]

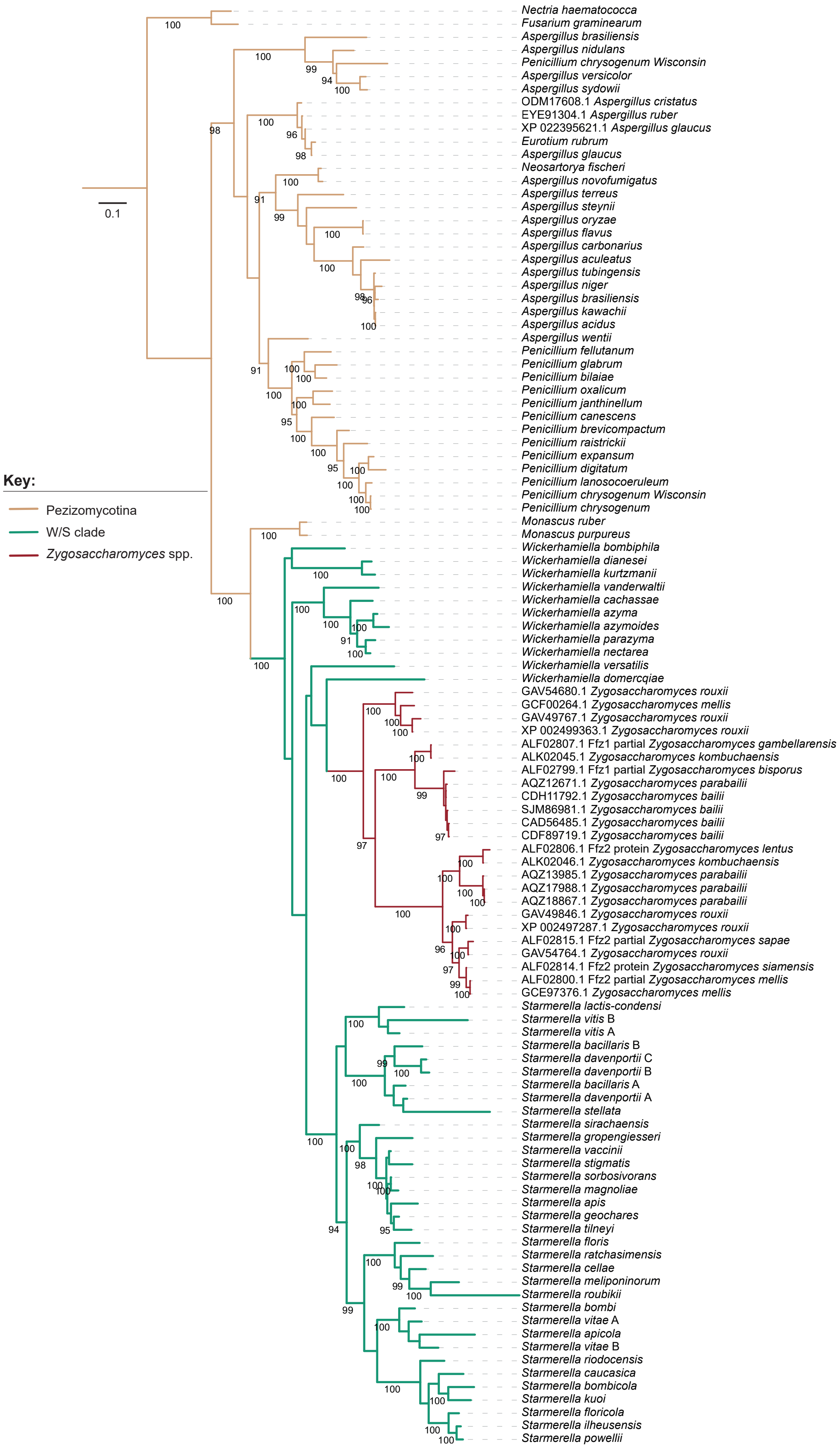

Supplementary Figure S7. Maximum-likelihood phylogeny of the Ffz1 transporter. Phylogeny depicting the relationships between W/S clade Ffz1 proteins and their closest relatives. The different lineages are represented by different branch colors (red for *Zygosaccharomyces* spp., light orange for other Pezizomycotina and blue for W/S clade) as indicated in the key. Bootstrap support values are indicated below the respective branch.
