# Supplementary material for: Extensive remodeling of sugar metabolism through gene loss and horizontal gene transfer in a eukaryotic lineage
Source: BMC Biol. 2024 May 30;22:128. doi: 10.1186/s12915-024-01929-7 (PMC11140947; doi:10.1186/s12915-024-01929-7)
Supplement: Supplementary file 11 — Additional file 11: Table S4. Kinetic parameters of hexokinase activity for seven W/S clade species with different trait combinations. No competitive experiments were performed, which might reveal additional properties of hexokinases in the various species. [file 12915_2024_1929_MOESM11_ESM.docx]

**Supplementary** **Table S4 - Kinetic parameters of hexokinase activity for seven W/S clade species with different trait combinations.** No competitive experiments were performed, which might reveal additional properties of hexokinases in the various species.

| Species | GLUCOSE | | FRUCTOSE | | K_m_ Ratio | V_max_ Ratio |
| --- | --- | --- | --- | --- | --- | --- |
|  | K_m_ (mM) | V_max_ (nM/sec) | K_m_ (mM) | V_max_ (nM/sec) |  |  |
| *W. domerqiae* | 0.43 ± 0.06 | 7.51 ± 0.33 | 1.79 ± 0.32 | 13.69 ± 0.61 | 4.17 | 1.822 |
| *W. versatilis* | 0.18 ± 0.04 | 4.75 ± 0.20 | 2.84 ± 0.53 | 4.51 ± 0.23 | 15.42 | 0.95 |
| *W. alocasiicola* | 0.21 ± 0.06 | 20.81 ± 1.3 | 2.24 ± 2.27 | 10.77 ± 2.80 | 10.58 | 0.52 |
| *W. australiensis* | 0.16 ± 0.02 | 26.91 ± 0.73 | 1.27 ± 0.35 | 36.13 ± 2.26 | 7.81 | 1.34 |
| *W. jalapaonensis* | 0.53 ± 0.29 | 4.15 ± 0.58 | 2.79 ± 3.40 | 4.88 ± 1.59 | 5.22 | 1.17 |
| *W. nakhonpathomensis* | 0.21 ± 0.04 | 6.53 ± 0.26 | 1.34 ± 0.98 | 7.35 ± 1.24 | 6.49 | 1.13 |
| *W. cachassae* | 0.27 ± 0.02 | 64.24 ± 1.44 | 0.62 ± 0.14 | 62.08 ± 2.83 | 2.27 | 0.97 |

Hexokinase specific activities were determined using the following mathematical formula:

$$Activity= \frac{(sample slope-Baseline slope)}{\varepsilon(NADPH)}$$

$$Specific Activity= \frac{(Activity)}{\left[ Protein \right]in extract\times\frac{Volume of extract used}{Reaction final volume}}$$

ɛ (NADPH) = 6,220 M^-1^cm^-1^
